# Supplementary material for: Are socioeconomic inequalities in the incidence of small-for-gestational-age birth narrowing? Findings from a population-based cohort in the South of England
Source: BMJ Open. 2019 Jul 29;9(7):e026998. doi: 10.1136/bmjopen-2018-026998 (PMC6678068; doi:10.1136/bmjopen-2018-026998)

**Supplementary Table 1 – Full results of model 4 in Table 2. Risk of being born Small for Gestational Age (birthweight <10^th^ percentile for gestational age) by maternal socioeconomic indicator in the University Hospital Southampton (UHS) maternity population-based cohort (singleton live births 2004-2016)**

|  | OR | 99% CI | | p |
| --- | --- | --- | --- | --- |
| ***Highest qualification (ref = Degree)*** |  |  |  |  |
| College qualification | 1.108 | 1.002 | 1.225 | 0.009 |
| Secondary school or lower qualification | 1.319 | 1.186 | 1.466 | <0.001 |
| Maternal unemployment | 1.268 | 1.163 | 1.383 | <0.001 |
| Lone mother | 1.055 | 0.929 | 1.198 | 0.280 |
| Gestational Diabetes | 0.903 | 0.703 | 1.159 | 0.293 |
| Gestational Hypertension | 1.986 | 1.595 | 2.474 | <0.001 |
| Systolic blood pressure | 0.997 | 0.994 | 1.001 | 0.035 |
| Multiparous | 0.474 | 0.438 | 0.513 | <0.001 |
| Maternal age at booking | 1.014 | 1.006 | 1.021 | <0.001 |
| ***Maternal ethnicity (ref = White)*** |  |  |  |  |
| Mixed | 1.310 | 0.958 | 1.793 | 0.026 |
| Asian | 2.610 | 2.275 | 2.993 | <0.001 |
| Black/African/Caribbean | 1.784 | 1.394 | 2.282 | <0.001 |
| Chinese | 0.721 | 0.430 | 1.211 | 0.105 |
| Other | 1.453 | 1.099 | 1.922 | 0.001 |
| Not known | 1.153 | 0.985 | 1.350 | 0.020 |
| ***Maternal BMI (ref = Normal weight 18.5-24.9)*** |  |  |  |  |
| <18.5 (underweight) | 1.727 | 1.458 | 2.045 | <0.001 |
| 25-29.9 (overweight) | 0.763 | 0.697 | 0.835 | <0.001 |
| 30+ (obese) | 0.710 | 0.633 | 0.796 | <0.001 |
| **Maternal smoking (ref = Never smoked)** |  |  |  |  |
| Ex-smoker | 0.954 | 0.867 | 1.048 | 0.196 |
| Up to 10 cigarettes per day | 2.244 | 1.992 | 2.528 | <0.001 |
| 10-20 cigarettes per day | 2.894 | 2.511 | 3.335 | <0.001 |
| >20 cigarettes per day | 3.744 | 2.586 | 5.420 | <0.001 |
| Constant | 0.094 | 0.061 | 0.145 | <0.001 |
| n births = 64,535, n mothers = 43,787. OR = odds ratio; CI = confidence interval. In all models the standard errors are adjusted for multiple births per mother. | | | | |

**Supplementary Table 2 – Full results of model 4 in Table 3. Risk of being born Small for Gestational Age (birthweight <10^th^ percentile for gestational age) by maternal socioeconomic indicator in the University Hospital Southampton (UHS) maternity population-based cohort (singleton live births 2004-2016)**

|  | OR | 99% CI | | p |
| --- | --- | --- | --- | --- |
| ***Highest qualification (ref = Degree)*** |  |  |  |  |
| College qualification | 1.098 | 0.990 | 1.217 | 0.020 |
| Secondary school or lower qualification | 1.303 | 1.168 | 1.453 | <0.001 |
| Maternal unemployment | 1.237 | 1.128 | 1.356 | <0.001 |
| Partner unemployment | 1.270 | 1.128 | 1.431 | <0.001 |
| Gestational Diabetes | 0.919 | 0.711 | 1.186 | 0.392 |
| Gestational Hypertension | 2.066 | 1.650 | 2.587 | <0.001 |
| Systolic blood pressure | 0.997 | 0.994 | 1.001 | 0.057 |
| Multiparous | 0.472 | 0.435 | 0.512 | <0.001 |
| Maternal age at booking | 1.015 | 1.007 | 1.023 | <0.001 |
| ***Maternal ethnicity (ref = White)*** |  |  |  |  |
| Mixed | 1.280 | 0.926 | 1.769 | 0.050 |
| Asian | 2.603 | 2.263 | 2.994 | <0.001 |
| Black/African/Caribbean | 1.713 | 1.315 | 2.232 | <0.001 |
| Chinese | 0.684 | 0.400 | 1.171 | 0.069 |
| Other | 1.435 | 1.074 | 1.918 | 0.001 |
| Not known | 1.182 | 1.005 | 1.391 | 0.008 |
| ***Maternal BMI (ref = Normal weight 18.5-24.9)*** |  |  |  |  |
| <18.5 (underweight) | 1.768 | 1.482 | 2.108 | <0.001 |
| 25-29.9 (overweight) | 0.763 | 0.695 | 0.839 | <0.001 |
| 30+ (obese) | 0.695 | 0.616 | 0.783 | <0.001 |
| **Maternal smoking (ref = Never smoked)** |  |  |  |  |
| Ex-smoker | 0.949 | 0.861 | 1.047 | 0.169 |
| Up to 10 cigarettes per day | 2.217 | 1.956 | 2.512 | <0.001 |
| 10-20 cigarettes per day | 2.871 | 2.464 | 3.344 | <0.001 |
| >20 cigarettes per day | 3.326 | 2.192 | 5.046 | <0.001 |
| Constant | 0.088 | 0.056 | 0.138 | <0.001 |
| n births = 60,385, n mothers = 41,841. OR = odds ratio; CI = confidence interval. In all models the standard errors are adjusted for multiple births per mother. | | | | |

**Supplementary Table 3 –Risk of being born Small for Gestational Age (birthweight <10^th^ percentile for gestational age) by place of residence and maternal socioeconomic indicator in the University Hospital Southampton (UHS) maternity population-based cohort (singleton live births 2004-2016)**

| Socioeconomic factor | Sample | OR | | 99% CI | p |
| --- | --- | --- | --- | --- | --- |
| Mothers with a college qualification vs university degree [1] | All | 1.11 | 1.00 - 1.22 | | 0.009 |
|  | Southampton | 1.10 | 0.95 - 1.28 | | 0.092 |
| Mothers with a secondary school qualification vs university degree [1] | All | 1.32 | 1.19 - 1.47 | | <0.001 |
|  | Southampton | 1.29 | 1.11 - 1.51 | | <0.001 |
| Maternal unemployment at the first antenatal appointment vs employed [1] | All | 1.27 | 1.16 - 1.38 | | <0.001 |
|  | Southampton | 1.38 | 1.23 - 1.56 | | <0.001 |
| Lone motherhood at the first antenatal appointment vs partnered status [1] | All | 1.05 | 0.93 - 1.20 | | 0.280 |
|  | Southampton | 1.02 | 0.86 - 1.20 | | 0.757 |
| Mothers with an unemployed partner vs employed partner | All | 1.27 | 1.13 - 1.43 | | <0.001 |
|  | Southampton | 1.19 | 1.03 - 1.39 | | 0.003 |
| All models adjusted for maternal education, employment, age, ethnicity, parity, gestational diabetes, gestational hypertension and systolic blood pressure. Standard errors are adjusted for multiple births per mother. [1] also adjusted for maternal partnership. OR = odds ratio; CI = confidence interval. | | | | | |

Supplementary Figure 1: Sample selection flowchart for the University Hospital Southampton cohort analysis of socioeconomic inequalities in small for gestational age births


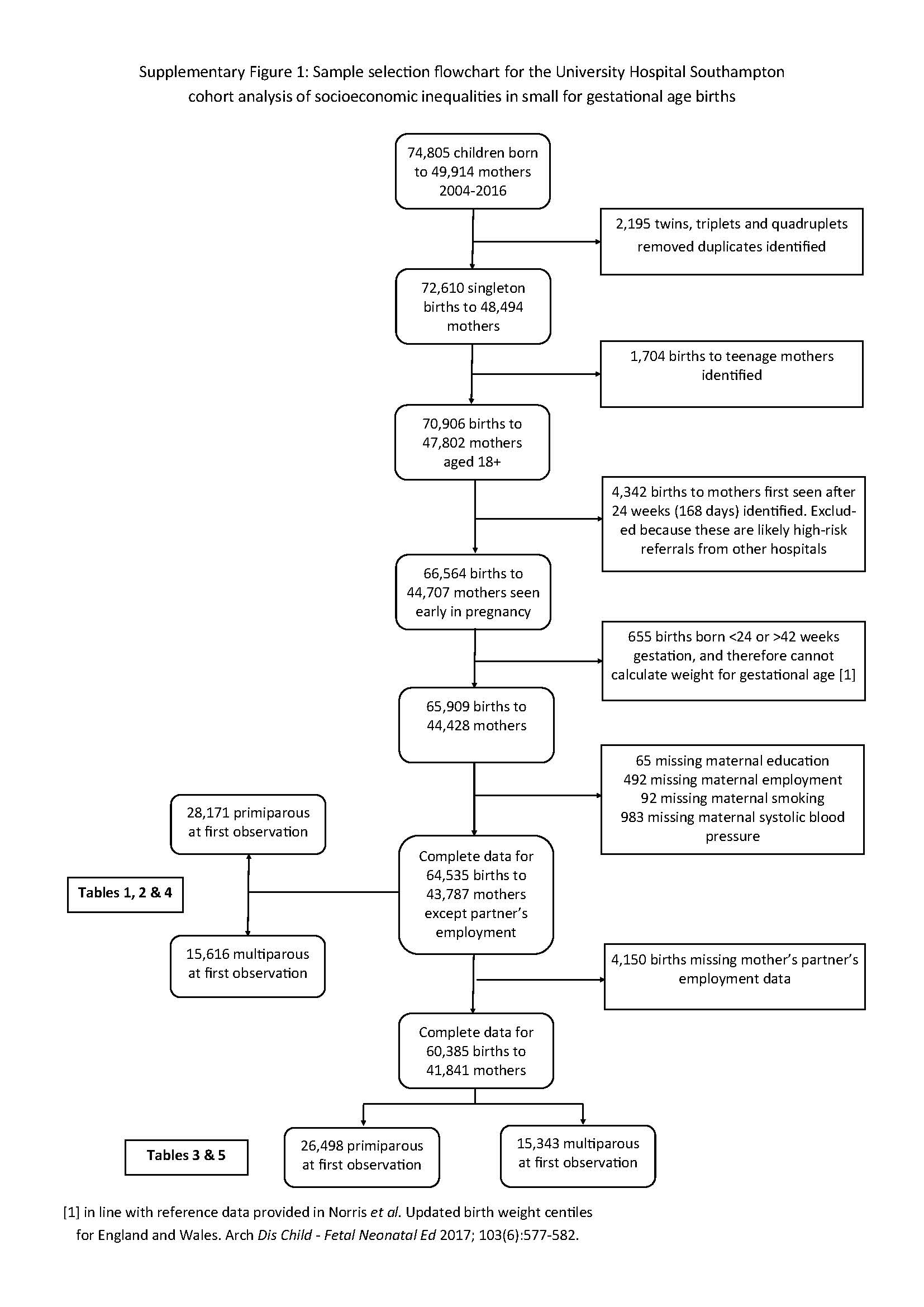


Supplementary Figure 2: Trends in socioeconomic factor over time in the University Hospital Southampton cohort analysis of socioeconomic inequalities in small for gestational age births


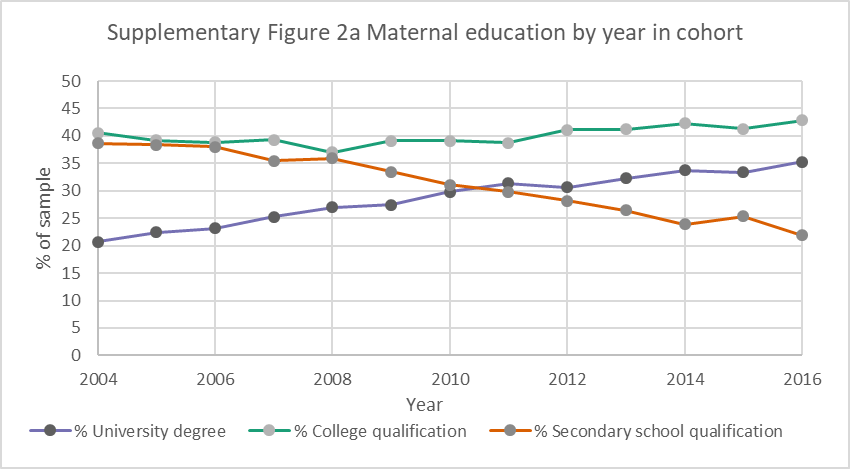


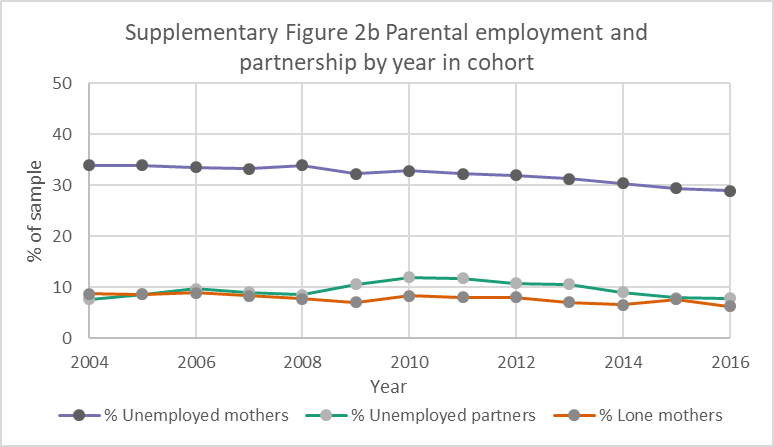

Supplement: Supplementary data [file bmjopen-2018-026998supp001.docx]
