# Supplementary material for: Are socioeconomic inequalities in the incidence of small-for-gestational-age birth narrowing? Findings from a population-based cohort in the South of England
Source: BMJ Open. 2019 Jul 29;9(7):e026998. doi: 10.1136/bmjopen-2018-026998 (PMC6678068; doi:10.1136/bmjopen-2018-026998)
Supplement: Author's manuscript [file bmjopen-2018-026998.draft_revisions.pdf]

BMJ Open is committed to open peer review. As part of this commitment we make the peer review history of every article we publish publicly available.

When an article is published we post the peer reviewers' comments and the authors' responses online. We also post the versions of the paper that were used during peer review. These are the versions that the peer review comments apply to.

The versions of the paper that follow are the versions that were submitted during the peer review process. They are not the versions of record or the final published versions. They should not be cited or distributed as the published version of this manuscript.

BMJ Open is an open access journal and the full, final, typeset and author-corrected version of record of the manuscript is available on our site with no access controls, subscription charges or pay-per-view fees (http://bmjopen.bmj.com).

If you have any questions on BMJ Open's open peer review process please email info.bmjopen@bmj.com

BMJ Open

Are socioeconomic inequalities in the incidence of small-for-gestational-age birth narrowing? Findings from a population-based cohort in the South of England

Journal:	*BMJ Open*
Manuscript ID	bmjopen-2018-026998
Article Type:	Research
Date Submitted by the Author:	01-Oct-2018

Complete List of Authors:	Wilding, Sam; University of Southampton, Academic Unit of Primary Care and Population Sciences, Faculty of Medicine<br>Ziauddeen, Nida; University of Southampton, Academic Unit of Primary Care and Population Sciences, Faculty of Medicine<br>Roderick, Paul; University of Southampton, Academic Unit of Primary Care and Population Sciences, Faculty of Medicine<br>Smith, Dianna; University of Southampton, Geography & Environment<br>Chase, Debbie; Southampton City Council, Public Health<br>Macklon, Nick; University of Copenhagen, Department of Obstetrics and Gynaecology; London Women's Clinic<br>McGrath, Nuala; University of Southampton, Academic Unit of Primary Care and Population Sciences, Faculty of Medicine; University of Southampton, School of Economic, Social and Political Science, Faculty of Social Sciences,<br>Hanson, Mark; University of Southampton, Institute of Developmental Sciences, Academic Unit of Human Development and Health, Faculty of Medicine; University of Southampton and University Hospital Southampton NHS Foundation Trust , NIHR Southampton Biomedical Research Centre<br>Alwan, Nisreen; University of Southampton, Academic Unit of Primary Care and Population Sciences, Faculty of Medicine; University of Southampton and University Hospital Southampton NHS Foundation Trust, NIHR Southampton Biomedical Research Centre

Keywords:	PUBLIC HEALTH, SOCIAL MEDICINE, small for gestational age, socioeconomic inequalities

SCHOLARONE™
Manuscripts

Are socioeconomic inequalities in the incidence of small-for-gestational-age birth narrowing? Findings from a population-based cohort in the South of England

Authors:

Sam Wilding[1], Nida Ziauddeen[1], Paul Roderick[1], Dianna Smith[2], Debbie Chase[3], Nicholas Macklon[4,5], Nuala McGrath[1,6], Mark Hanson[7,8], Nisreen A Alwan[1,8]

Author affiliations:

[1] Academic Unit of Primary Care and Population Sciences, Faculty of Medicine, University of Southampton

[2] Geography and Environmental Science, Faculty of Environmental and Life Sciences, University of Southampton

[3] Public Health, Southampton City Council

[4] Department of Obstetrics and Gynaecology, University of Copenhagen, Zealand University Hospital, Roskilde 4000, Denmark

[5] London Women's Clinic, 113-115 Harley Street, London W1G 6AP

[6] School of Economic, Social and Political Science, Faculty of Social Sciences, University of Southampton

[7] Institute of Developmental Sciences, Academic Unit of Human Development and Health, Faculty of Medicine, University of Southampton

[8] NIHR Southampton Biomedical Research Centre, University of Southampton and University Hospital Southampton NHS Foundation Trust

For peer review only

Abstract

Objectives

To investigate socioeconomic inequalities, using maternal educational attainment, maternal and partner employment status, and lone motherhood indicators, in the risk of small for gestational age (SGA) births, their time trend, potential mediation by maternal smoking and body mass index (BMI), and effect modification by parity.

Design

Population-based birth cohort utilising routine antenatal healthcare data.

Setting

Babies born at University Hospital Southampton, UK, between 2004 and 2016.

Participants

65,825 singleton live births born to mothers aged ≥18 years between 23 and 42 weeks gestation.

Main outcome measures

SGA (birth weight <10th percentile for others born at the same number of completed weeks compared to 2013/2014 within England and Wales).

Results

Babies born to mothers with no university degree (adjusted Odds Ratio (aOR) 1.19, 99% Confidence Interval (CI) 1.09-1.31), who were unemployed (aOR 1.31, CI 1.20-1.42) or with unemployed partners (aOR 1.31, CI 1.17-1.48) were at greater risk of being SGA. There was no statistically significant change in the magnitude of this risk difference by these indicators over time between 2004 and 2016, as estimated by linear interactions with year of birth. Babies born to lone mothers were not at higher risk compared to partnered mothers after adjusting for maternal smoking (aOR 1.08, CI 0.95-1.22). The inverse association between maternal educational attainment and SGA risk appeared greater in multiparous (aOR 1.29, CI 1.04-1.61) compared to primiparous women (aOR 1.16, CI 1.04-1.30), and the reverse was true for maternal unemployment where the association was stronger in primiparous women.

Conclusions

SES Inequalities in SGA risk by educational attainment and employment status are not narrowing over time, with differences in association strength by parity. The greater SGA risk in lone mothers was potentially explained by maternal smoking. Preventive interventions should target socially disadvantaged women, including preconception and postpartum smoking cessation to reduce SGA risk.

For peer review only

Strengths and limitations of this study

- This study uses routine data for all pregnancies in a regional specialist antenatal hospital to predict the risk of small-for-gestational-age births to mothers by socio-economic factors.
- Standard measures are used which can be used for risk prediction in practice without additional time required in antenatal appointments, given that there is no evidence of change in socioeconomic risk factors over time.
- Limitations include the transferability of results from this hospital to centres with differing populations, that socioeconomic factors were only measured at one time point, the exclusion of teenage mothers and late antenatal bookings, and self-reporting of educational qualifications and employment.

For peer review only - http://bmjopen.bmj.com/site/about/guidelines.xhtml

Introduction

Babies born small for gestational age (defined as <10th percentile for birthweight at a particular gestational age; SGA) are at higher risk of neonatal mortality [1] and childhood obesity through compensatory early growth [2]. A number of clinical and lifestyle risk factors are associated with the risk of being SGA, including maternal height, weight, diet, ethnicity, parity, smoking, pre-eclampsia and hypertension [3,4]. Beyond these risk factors, and closely linked to them, there is extensive evidence of Socio Economic Status (SES) inequalities, with babies born to mothers living in the most socioeconomically deprived areas being between 28% and 34% more likely to be born SGA than those in the most affluent quintile [5].

Several proxies of SES are present in the literature, with area measures of wealth, maternal education, socioeconomic position of maternal employment and income being the most common indicators, with paternal factors being notably absent [6]. The majority of studies rely on one proxy of SES, but studies controlling for several SES proxies find that different aspects of SES are independently associated with the risk of SGA [7–11]. Despite the wealth of research on the association between parental SES and SGA, the mechanisms underlying this association are poorly understood [12]. Current explanations focus on the availability of (physiological and material) resources and mediating factors that differ between women of high and low SES. For resources, the 'weathering' hypothesis states that women in low SES at the time of conception have experienced relatively high levels of cumulative disadvantage in terms of income, stress and diet, which have led to a deterioration in physiological health [13]. This association may also be mediated by lifestyle factors, wherein mothers in low SES are more likely to be exposed to or partake in risk factors for SGA, which in turn increase their relative risk of SGA. Mediation analyses have found that higher rates of underweight and smoking at conception among mothers with low educational attainment mediates the association between SES and birth outcomes in the UK [12,14]. The extent of these SES inequalities in the risk of SGA may differ between first and higher order births. The birth of the first child brings significant physiological, wellbeing and social changes [15], and women in low SES may have weaker social support mechanisms to adjust to these changes, as they appear to be at high risk of SGA in subsequent births after adjusting for clinical risk factors [16]. Risk factors for SGA specific to second and higher order births are more prevalent in women of low SES, with postnatal depression being more common in mothers without a university degree and those in poverty [17,18].

Public health policy aims to narrow SES inequalities in birth outcomes over time, and there is reason to believe that inequalities in SGA may have changed since the early-2000s. Major welfare reforms enacted in the UK between 1999 and 2002 increased in-work tax incentives, which particularly increased the net income of part-time working women, relative to those out of work [19]. In 2008 the global 'great recession' occurred, after which single mothers in England became increasingly less likely to be employed, whilst facing disproportionate losses of welfare income, facing a double income penalty relative to working mothers [20]. The recession appears to have had differential impacts on women by level of educational attainment, with those without a university degree experiencing a post-recession rise in the prevalence of obesity, relative to those with degrees [21].

Utilising a maternity healthcare database in Hampshire, England, we aimed to examine differences in SGA risk by SES indicators, investigate if these differences are mediated by maternal weight and smoking, and whether they have narrowed over the 13 year study period (2004-2016). In addition, we aimed to stratify by parity in order to examine whether the extent of SES inequalities are the same at first births, relative to 2nd and higher order births.

For peer review only - http://bmjopen.bmj.com/site/about/guidelines.xhtml

Methods

Data

This analysis is based on a population-based cohort including anonymised antenatal and delivery records of women aged ≥18 years who had a live singleton birth between 1 January 2004 and 31 December 2016 at the University Hospital Southampton (UHS) National Health Service (NHS) Trust in the South of England. UHS is the primary centre for maternity care for the city of Southampton and the surrounding areas, and is the regional centre for high-risk pregnancies. To ensure that the findings are applicable to the majority of (non high-risk) pregnancies, records with late first antenatal (booking) appointments (after 24 weeks gestation, as assessed by ultrasound) and of mothers under the age of 18 were excluded. First, we analysed the risk of SGA by SES in all births (including more than one birth per mother if in the database and study timeframe), adjusting for confounding and clustering. We then tested whether differences between SES groups (by maternal education, employment, paternal employment and partnership status) have changed over the study period (2004-2016). We then limited the analysis to the first recorded birth per mother in the dataset, and stratified by parity (primiparous and multiparous), to avoid biasing sub-analyses via double-counting. This project was approved by the University of Southampton Faculty of Medicine Ethics Committee (ref 24433) and the NHS Health Research Authority (ref 242031).

Assessment of SES exposures

Socioeconomic measures were self-reported at the first antenatal (booking) appointment, which is recommended by the National Institute for Health and Care Excellence (NICE) Antenatal Care Guidelines to occur by the 10th week of gestation [22]. Mothers were asked to report their highest educational qualification, whether they were currently employed, and if their partners were currently employed (possible answers included employed, unemployed and seeking work or student). Partnership status was self-reported at the same appointment. All four SES proxies were dichotomised, with values of 1 indicating lower SES (mother does not have a degree; mother is unemployed; mother's partner is unemployed; mother is single).

Assessment of outcome

Birth weight was measured by healthcare professionals for all births in the dataset. Gestational age was based on a dating ultrasound scan performed by healthcare professionals, and was present for all records in the dataset. Birth weight centile for gestational age is calculated using reference values provided in the most recently released data (2013-2014) for England and Wales, which were validated using 2015 records [23]. Given that the association between SES and preterm births is well established in the literature [24], and that gestational age is strongly associated with birthweight, we use a Small for Gestational Age (SGA) measure to assess low birth weight rather than the standard birth weight cut-off.

The birth centile references are available for 24-42 completed weeks of gestation, so live births at ≤23 (71) or >42 (564) completed weeks or with indeterminate sex (16) are excluded from the analysis (SGA sample = 65,825/66,476). SGA is defined as a birth weight lower than the 10th percentile compared to others born at the same number of weeks gestation in the sex-specific reference centiles [23], and all others are defined as Not Small for Gestational Age (non-SGA).

Assessment of confounder and mediator variables

Maternal age, height, smoking history (never smoked, ex-smoker and current smoker at the time of the booking appointment), parity and ethnicity were self-reported at the booking appointment. Baby's sex was assessed at birth by a healthcare professional. Maternal weight and blood pressure were measured by a healthcare professional at the booking appointment. Maternal age, ethnicity

For peer review only - http://bmjopen.bmj.com/site/about/guidelines.xhtml

and systolic blood pressure were adjusted for in the multivariable models, as these factors have been associated with the risk of SGA in previous analyses [3,25,26]. Parity (no versus 1 or more previous births) was treated as a confounder in the models analysing the whole sample, and then as an effect modifier for SES through interaction terms and later stratification. Maternal body mass index (BMI) and smoking history are included as potential mediators of the relationship between SES and risk of SGA, based on previous evidence [12,14].

Statistical analysis

All analyses were conducted using Stata 15 (College Station, Texas). Descriptive statistics and the unadjusted odds ratios (ORs) between all variables and risk of SGA are presented in Table 1. T-tests were used to test whether the mean of each continuous variable (maternal BMI, age and systolic blood pressure at booking) differed between those born SGA and non-SGA. Multivariable logistic regression models were used to estimate ORs, p-values and respective 99% confidence intervals (CI) for SES differences in the risk of SGA independently after adjustment for control variables, after adjustment for other SES indicators, and then after controlling for mediators. A p-value cut-off of 0.01 is used to test for statistical significance when reporting risk rather than the more conventional 0.05 cut-off in order to minimise the risk of type I error due to multiple testing, as adjusted models control for multiple SES indicators [27]. Evidence of mediation is examined through assessing the attenuation of SES with SGA associations once known risk factors are controlled for, and the significance once each a priori mediator (first BMI, then smoking) is controlled for [28]. In all logistic regressions, cases with missing data for variables within the model were dropped (complete case analysis).

In the first analysis, adjusted ORs for the risk of a baby being born SGA are presented in model 1 (control variables include maternal age, parity, ethnicity, and systolic blood pressure at booking) independently for maternal education, employment and partnership status, adjusting for clustering of births within the same mother. In model 2, all three of these SES proxies are controlled for, in additional to the control variables in model 1, before including the two mediators (maternal BMI and smoking) sequentially in models 3 and 4. Due to collinearity between maternal partnership and partner's employment, the association for the latter is tested separately with the same structure.

In the second analysis, year and the interactions between year and SES indicator (slope) effects are included to model 4 for maternal education, employment, partner's employment and partnership status, to test whether SES inequalities in the risk of being born SGA are widening or narrowing over time during the study period. These slopes represent the change in relative odds of SGA for the socioeconomic group relative to the control group for each year in the dataset (2004-2016). Odds ratios >1 indicate that this group became at higher risk of SGA births over time, relative to the control group [29]. Further models were estimated including SES interactions between a dummy indicator for records pre- (2004-2008) and post- (2009-2016) 2008, to test whether SES inequalities in the risk of SGA changed in magnitude between the two periods.

In the third analysis, the sample is limited to the first birth for each mother (1 birth per mother), and then stratified by parity (primiparous or multiparous). Limiting the sample to the first birth for each mother acts as a sensitivity analysis for the first analysis, ensuring that the results are not influenced by multiple births per mother. Interactions between SES and parity are estimated to test whether the association between SES and risk of SGA is modified by parity, and then parity-stratified modelling was conducted. A p-value cut-off of 0.05 is used to test for interactions. As in the first analysis, adjusted SES ORs are presented for each sub-sample, then these ORs are adjusted for other SES indicators, before including mediators (maternal BMI and smoking).

For peer review only - http://bmjopen.bmj.com/site/about/guidelines.xhtml

Results

There are 65,825 singleton live births within the dataset which can be categorised as SGA or non-SGA to 44,371 mothers. Of births, 71% were to women with no university degree, in employment (67.9%), have partners the time of booking (92.3%), who are in employment (90.4%), of white ethnicity (82.4%) and with normal (<140 mm Hg) systolic blood pressure (98.7%). Of these 65,825 births, 6,343 (9.6%, 99% CI 9.4%-9.9%) were born SGA (Table 1).

The proportion of SGA births was higher than the average for births to mothers in all disadvantaged SES groups. This includes births to mothers with no university degree (10.3% born SGA, 99% CI 9.9-10.6), births to unemployed mothers (11.6% born SGA, 99% CI 11.1-12.2), births to mothers with unemployed partners (14.2% born SGA, 99% CI 13.0-15.4), and births to single mothers (12.4% born SGA, 99% CI 11.3-13.7). Other maternal factors associated with a higher than average rate of SGA include maternal BMI <18.5 kg/m^2 (19.9% born SGA, 99% CI 17.6-22.3), maternal smoking at booking (16.8% born SGA, 99% CI 15.9-17.8) and Asian ethnicity (18.3% born SGA, 99% CI 16.8-19.8).

Table 1 – Maternal characteristics of all babies by Small for Gestational Age status (birthweight <10th percentile for gestational age) in the University Hospital Southampton (UHS) maternity population-based cohort (singleton live births 2004-2016, n=65,825)

	SGA		Non-SGA		% SGA[a]	
Maternal characteristics	n	(%)	n	(%)	% SGA	(99% CI)
Highest qualification						
University degree or higher	1,545	(24.4)	17,498	(29.5)	8.1	(7.6 - 8.6)
Lower than a university degree	4,795	(75.6)	41,924	(70.6)	10.3	(9.9 - 10.6)
Employment status						
Employed	3,868	(61.3)	40,511	(68.6)	8.7	(8.4 - 9.1)
Unemployed	2,438	(38.7)	18,519	(31.4)	11.6	(11.1 - 12.2)
Partner's employment status						
Employed	4,969	(85.6)	50,621	(90.9)	8.9	(8.6 - 9.3)
Unemployed	838	(14.4)	5,075	(9.1)	14.2	(13.0 - 15.4)
Partnership						
Partnered	5,706	(90.0)	54,994	(92.5)	9.4	(9.1 - 9.7)
Lone mother	637	(10.0)	4,488	(7.6)	12.4	(11.3 - 13.7)
BMI						
<18.5	393	(6.2)	1,586	(2.7)	19.9	(17.6 - 22.3)
18.5-24.9	3,628	(57.2)	30,722	(51.7)	10.6	(10.1 - 11)
25-29.9	1,425	(22.5)	16,070	(27.0)	8.1	(7.6 - 8.7)
30+	897	(14.1)	11,104	(18.7)	7.5	(6.9 - 8.1)
Smoking						
Never smoked	3,050	(48.1)	30,760	(51.8)	9.0	(8.6 - 9.4)
Ex-smoker	1,488	(23.5)	19,735	(33.2)	7.0	(6.6 - 7.5)
Current smoker	1,801	(28.4)	8,902	(15.0)	16.8	(15.9 - 17.8)
Age (years)						
18-24	2,000	(31.5)	14,343	(24.1)	12.2	(11.6 - 12.9)
25-34	3,420	(53.9)	35,641	(59.9)	8.8	(8.4 - 9.1)
35-39	754	(11.9)	7,998	(13.5)	8.6	(7.9 - 9.4)
40+	169	(2.7)	1,500	(2.5)	10.1	(8.3 - 12.2)
Previous live births						

For peer review only - http://bmjopen.bmj.com/site/about/guidelines.xhtml

Maternal characteristics	SGA n	SGA (%)	Non-SGA n	Non-SGA (%)	% SGA[a] % SGA	% SGA[a] (99% CI)
None	3,515	(55.4)	25,097	(42.2)	12.3	(11.8 - 12.8)
One or more	2,828	(44.6)	34,385	(57.8)	7.6	(7.2 - 8)
Ethnicity						
White	4,793	(75.6)	49,477	(83.2)	8.8	(8.5 - 9.2)
Mixed	87	(1.4)	720	(1.2)	10.8	(8.1 - 13.9)
Asian	809	(12.8)	3,621	(6.1)	18.3	(16.8 - 19.8)
Black/African/Caribbean	148	(2.3)	1,096	(1.8)	11.9	(9.6 - 14.4)
Chinese	31	(0.5)	427	(0.7)	6.8	(4.1 - 10.4)
Other	116	(1.8)	831	(1.4)	12.2	(9.6 - 15.2)
Not known	359	(5.7)	3,310	(5.6)	9.8	(8.6 - 11.1)
Systolic blood pressure at first antenatal appointment						
<140 mm Hg	6,275	(99.0)	58,578	(98.6)	9.7	(9.4 - 10)
>=140 mm Hg	64	(1.0)	812	(1.4)	7.3	(5.2 - 9.9)
**Overall**	6,343	(100)	59,482	(100)	9.6	(9.4 - 9.9)

	SGA Mean	SGA (SD)	Non-SGA Mean	Non-SGA (SD)	p-value for t-test
BMI	24.5	5.2	25.7	5.5	<0.001
Age	27.9	5.8	28.8	5.5	<0.001
Systolic blood pressure	107.5	16.2	108.4	17.0	<0.001

Source: UHS antenatal records for live singleton births to mothers ≥18 years (2004-2016). Records with a late antenatal booking (over 24 weeks gestation) were excluded. Variables with missing information include maternal education (63), maternal employment (489) and partner's employment (4,322). [a] The percentage of babies born SGA relative to non-SGA for this characteristic, and the accompanying 99% confidence interval. The t-test indicates whether the mean of each variable differs between those born SGA and non-SGA.

For peer review only

SES differences in SGA risk in the whole cohort

Estimates of the association between maternal SES indicators and risk of SGA are presented in Table 2. The univariable associations between each SES indicator and the risk of SGA are presented in the unadjusted risk row, with all SES indicators being associated with SGA. The size of these effects increase in the first adjusted model (controlling for maternal age, ethnicity, parity and systolic blood pressure), and attenuate once other SES indicators are controlled for (model 2). Accounting for maternal BMI increased educational differences (from an OR of 1.29 to 1.34), but did not affect the estimates for employment or partnership (model 3). After including maternal smoking all SES inequalities reduced in size (model 4), with the OR for lone motherhood attenuating to statistical insignificance at the 99% level (OR 1.08, 99% CI 0.95-1.22, p=0.133).

For peer review only - http://bmjopen.bmj.com/site/about/guidelines.xhtml

Table 2– Risk of being born Small for Gestational Age (birthweight <10th percentile for gestational age) by maternal socioeconomic indicator in the University Hospital Southampton (UHS) maternity population-based cohort (singleton live births 2004-2016).

	Maternal educational qualification less than a university degree			Maternal unemployment at the first antenatal appointment			Lone motherhood at the first antenatal appointment*		
	OR	99% CI	p	OR	99% CI	p	OR	99% CI	p
Unadjusted risk	1.30	(1.19 - 1.41)	<0.001	1.38	(1.28 - 1.49)	<0.001	1.37	(1.22 - 1.54)	<0.001
Adjusted risk - Model 1	1.36	(1.25 - 1.48)	<0.001	1.55	(1.42 - 1.68)	<0.001	1.41	(1.25 - 1.59)	<0.001
Adjusted risk - Model 2	1.29	(1.18 - 1.40)	<0.001	1.47	(1.35 - 1.60)	<0.001	1.27	(1.13 - 1.44)	<0.001
Adjusted risk - Model 3	1.34	(1.23 - 1.47)	<0.001	1.48	(1.36 - 1.61)	<0.001	1.27	(1.13 - 1.44)	<0.001
Adjusted risk - Model 4	1.19	(1.09 - 1.31)	<0.001	1.31	(1.20 - 1.43)	<0.001	1.08	(0.95 - 1.22)	0.133

#Model 1 adjusts for maternal age, ethnicity, parity and systolic blood pressure.

Model 2 is model 1 plus the other two SES indicators (n births = 65,331, n mothers = 44,158).

###Model 3 is model 2 plus maternal body mass index (continuous) as a potential mediator (n births = 65,331, n mothers = 44,158).

####Model 4 is model 3 plus maternal smoking history (categorical) as an additional mediator (n births = 65,331, n mothers = 44,158).

OR = odds ratio; CI = confidence interval. In all models the standard errors are adjusted for multiple births per mother.

For peer review only

For peer review only - http://bmjopen.bmj.com/site/about/guidelines.xhtml

In unadjusted estimates, those born to mothers with unemployed partners at the antenatal booking appointment are 68% more likely to be born SGA (OR 1.68 99% CI 1.51-1.88) in comparison to those born to mothers with employed partners. This association attenuates once confounders are controlled for (model 1), but extenuates once maternal education and employment are controlled for (model 2). The association attenuates further once maternal BMI is controlled for (model 3) and remains similar once smoking is accounted for (model 4 OR 1.31, 99% CI 1.17-1.48, p <0.001) (Table 3).

For peer review only

For peer review only - http://bmjopen.bmj.com/site/about/guidelines.xhtml

Table 3 – Risk of being born Small for Gestational Age (birthweight <10th percentile for gestational age) by partner's employment status in the University Hospital Southampton (UHS) maternity population-based cohort (singleton live births 2004-2016).

	Mothers with unemployed partners at the first antenatal appointment		
	OR	99% CI	p
Unadjusted risk	1.68	(1.51 - 1.88)	<0.001
Adjusted risk - Model 1	1.69	(1.51 - 1.89)	<0.001
Adjusted risk - Model 2	1.51	(1.35 - 1.69)	<0.001
Adjusted risk - Model 3	1.55	(1.38 - 1.74)	<0.001
Adjusted risk - Model 4	1.31	(1.17 - 1.48)	<0.001

#Model 1 adjusts for maternal age, ethnicity, parity and systolic blood pressure.

Model 2 is model 1 plus the other two SES indicators (n births = 61,170, n mothers = 42,217).

###Model 3 is model 2 plus maternal body mass index as a potential mediator (n births = 61,170, n mothers = 42,217).

####Model 4 is model 3 plus maternal smoking history as an additional mediator (n births = 61,170, n mothers = 42,217).

OR = odds ratio; CI = confidence interval. In all models the standard errors are adjusted for multiple births per mother.

For peer review only

Time trend in SES inequalities in the risk of SGA between 2004 and 2016

To test whether SES inequalities are narrowing or widening over time, interactions between year (continuous) and SES ('slope') were included to model 4 in Table 2 and Table 3, and expressed as ORs. A positive slope OR indicates that the disadvantaged SES group are becoming at greater risk of SGA relative to the advantaged group over calendar year, and vice versa for a negative effect.

Figure 1a-d displays the adjusted ORs for each SES indicator by year in the cohort (UHS), and the accompanying p-value for the slope over calendar year. The slopes for maternal education (OR 1.00, 99% CI 0.98-1.02), lone motherhood (OR 1.00, 99% CI 0.97-1.03) and partner unemployment (OR 1.00, 99% CI 0.97-1.03) were negative but not statistically significant, whilst the slope for maternal employment (OR 1.00, 99% CI 0.98-1.02) was positive but also not statistically significant. Models using a binary indicator for pre- and post-2008 (2003-2008 and 2009-2016) showed no significant differences in the magnitude of SES inequalities (results not shown).

For peer review only

For peer review only - http://bmjopen.bmj.com/site/about/guidelines.xhtml

Figure 1 - Risk of being born Small for Gestational Age (birthweight <10th percentile for gestational age) by parental SES indicators in the University Hospital Southampton (UHS) maternity population-based cohort (singleton live births 2004-2016).

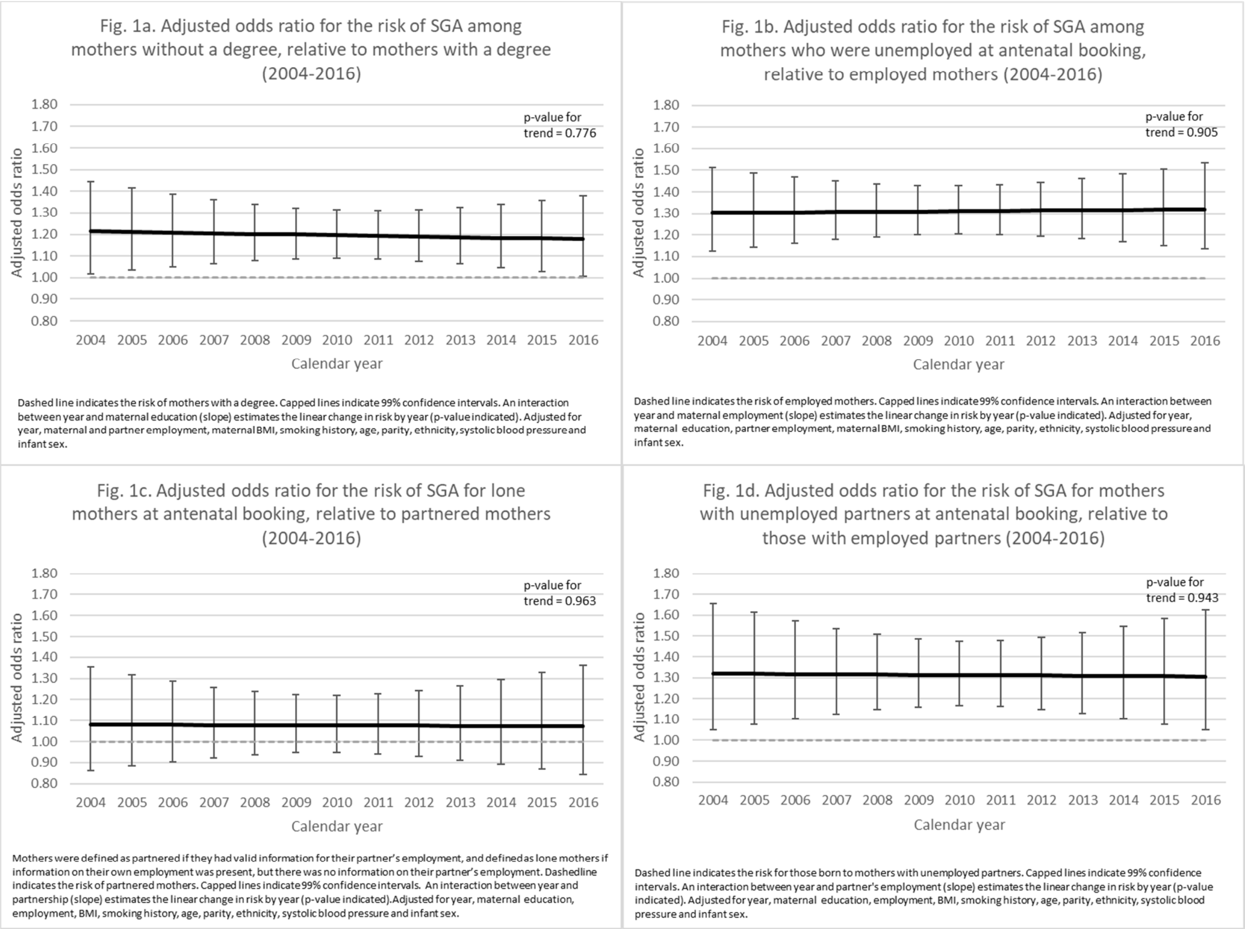

For peer review only - http://bmjopen.bmj.com/site/about/guidelines.xhtml

SES differences in SGA risk by maternal parity status

For this analysis, the sample was restricted to the first antenatal care record per mother included in our dataset with no missing information (21,667 records dropped, with a new total of 44,158). Interaction terms between each SES indicator and parity (accounting for control variables) were conducted utilising this sample showing a near significant interaction between maternal educational qualification and SGA (p = 0.06), and a significant interaction between maternal employment status and SGA (p=0.01). We then stratified the sample by parity (n primiparous (0 previous live births) = 28,469; n multiparous (1 or more previous live births) = 15,699). The modelling strategy used in the first analysis is repeated on these sub-samples to assess the risk estimates by parity.

The association between maternal education and risk of SGA appeared less pronounced among primiparous (aOR 1.16, 99% CI 1.04-1.30) than multiparous women (aOR 1.29, CI 1.04-1.61). Maternal unemployment (relative to mothers who were employed) was associated with higher risk of SGA in all samples, with a stronger association in the model limited to primiparous women (aOR 1.33, 99% CI 1.17-1.51) than in the model limited to multiparous women (aOR 1.21, 99% CI 1.03-1.42). The association between lone motherhood and SGA risk appeared to be mediated by smoking in all sub-samples (Table 4).

Table 5Table 5 displays the results for partner's employment (total n mothers = 42,217; 26,792 primiparous, 15,425 multiparous). The association between partner's employment and risk of SGA appeared to be mediated by maternal smoking among multiparous women (aOR 1.21, CI 0.98-1.49), but not primiparous women (aOR 1.35, CI 1.14-1.60). The estimates of SES differences in the risk of SGA were similar in the reduced sample (Tables 4 and 5) and the whole sample (Tables 2 and 3).

For peer review only - http://bmjopen.bmj.com/site/about/guidelines.xhtml

Table 4 - Risk of being born Small for Gestational Age (birthweight <10th percentile for gestational age) by maternal socioeconomic indicator and stratified by parity in the University Hospital Southampton (UHS) maternity population-based cohort (singleton live births 2004-2016, one live birth per mother)

		Maternal educational qualification less than a university degree			Maternal unemployment at the first antenatal appointment			Lone motherhood at the first antenatal appointment*		
	Model	OR	99% CI	p	OR	99% CI	p	OR	99% CI	p
Whole sample n mothers = 44,158	Unadjusted risk	1.22	(1.12 - 1.34)	<0.001	1.37	(1.26 - 1.49)	<0.001	1.33	(1.17 - 1.52)	<0.001
	Adjusted risk - Model 1#	1.31	(1.19 - 1.44)	<0.001	1.48	(1.35 - 1.63)	<0.001	1.40	(1.22 - 1.60)	<0.001
	Adjusted risk - Model 2##	1.27	(1.15 - 1.40)	<0.001	1.43	(1.29 - 1.57)	<0.001	1.28	(1.12 - 1.47)	<0.001
	Adjusted risk - Model 3###	1.32	(1.20 - 1.45)	<0.001	1.42	(1.29 - 1.56)	<0.001	1.29	(1.12 - 1.48)	<0.001
	Adjusted risk - Model 4####	1.19	(1.08 - 1.31)	<0.001	1.29	(1.16 - 1.42)	<0.001	1.11	(0.97 - 1.28)	0.055
Primiparous women only n births = 28,469	Unadjusted risk	1.25	(1.13 - 1.38)	<0.001	1.78	(1.59 - 1.99)	<0.001	1.32	(1.12 - 1.56)	<0.001
	Adjusted risk - Model 1#	1.24	(1.11 - 1.38)	<0.001	1.50	(1.32 - 1.69)	<0.001	1.33	(1.12 - 1.57)	<0.001
	Adjusted risk - Model 2##	1.21	(1.09 - 1.35)	<0.001	1.45	(1.28 - 1.64)	<0.001	1.22	(1.03 - 1.45)	0.003
	Adjusted risk - Model 3###	1.25	(1.12 - 1.39)	<0.001	1.44	(1.27 - 1.63)	<0.001	1.23	(1.03 - 1.46)	0.002
	Adjusted risk - Model 4####	1.16	(1.04 - 1.30)	0.001	1.33	(1.17 - 1.50)	<0.001	1.10	(0.92 - 1.31)	0.172
Multiparous women only n births = 15,699	Unadjusted risk	1.52	(1.24 - 1.86)	<0.001	1.52	(1.31 - 1.77)	<0.001	1.49	(1.19 - 1.85)	<0.001
	Adjusted risk - Model 1#	1.60	(1.29 - 1.97)	<0.001	1.44	(1.23 - 1.69)	<0.001	1.55	(1.23 - 1.94)	<0.001
	Adjusted risk - Model 2##	1.50	(1.21 - 1.85)	<0.001	1.35	(1.15 - 1.59)	<0.001	1.40	(1.11 - 1.76)	<0.001
	Adjusted risk - Model 3###	1.59	(1.28 - 1.97)	<0.001	1.37	(1.17 - 1.61)	<0.001	1.41	(1.12 - 1.77)	<0.001
	Adjusted risk - Model 4####	1.29	(1.04 - 1.61)	0.003	1.21	(1.03 - 1.42)	0.003	1.12	(0.89 - 1.42)	0.214

#Model 1 adjusts for maternal age, ethnicity and systolic blood pressure.

Model 2 is model 1 plus the other two SES indicators (e.g. the maternal education column is adjusted for maternal employment and partnership).

###Model 3 is model 2 plus maternal body mass index as a potential mediator.

####Model 4 is model 3 plus maternal smoking history as an additional mediator.

OR = odds ratio; CI = confidence interval. All models for the whole sample are adjusted for parity.

For peer review only - http://bmjopen.bmj.com/site/about/guidelines.xhtml

Table 5 - Risk of being born Small for Gestational Age (birthweight <10th percentile for gestational age) by partner's employment and stratified by parity in the University Hospital Southampton maternity population-based cohort (singleton live births 2004-2016)

		Unemployed partner at first antenatal appointment		
	Model	OR	99% CI	p
Whole sample n mothers = 42,217	Unadjusted risk	1.61	(1.42 - 1.81)	<0.001
	Adjusted risk - Model 1#	1.61	(1.42 - 1.82)	<0.001
	Adjusted risk - Model 2##	1.46	(1.28 - 1.66)	<0.001
	Adjusted risk - Model 3###	1.49	(1.31 - 1.70)	<0.001
	Adjusted risk - Model 4####	1.30	(1.14 - 1.49)	<0.001
Primiparous women only n births = 26,792	Unadjusted risk	1.75	(1.50 - 2.05)	<0.001
	Adjusted risk - Model 1#	1.59	(1.36 - 1.87)	<0.001
	Adjusted risk - Model 2##	1.45	(1.22 - 1.71)	<0.001
	Adjusted risk - Model 3###	1.47	(1.24 - 1.74)	<0.001
	Adjusted risk - Model 4####	1.35	(1.14 - 1.60)	<0.001
Multiparous women only n births = 15,425	Unadjusted risk	2.03	(1.37 - 2.03)	<0.001
	Adjusted risk - Model 1#	1.63	(1.33 - 1.99)	<0.001
	Adjusted risk - Model 2##	1.47	(1.20 - 1.81)	<0.001
	Adjusted risk - Model 3###	1.52	(1.24 - 1.87)	<0.001
	Adjusted risk - Model 4####	1.21	(0.98 - 1.49)	0.021

#Model 1 adjusts for parity, maternal age, ethnicity and systolic blood pressure.

Model 2 is model 1 plus maternal education and employment.

###Model 3 is model 2 plus maternal body mass index as a potential mediator.

####Model 4 is model 3 plus maternal smoking history as an additional mediator.

For peer review only

Discussion

In this analysis of routine maternity healthcare data from a regional hospital in Southampton, UK, multivariable logistic regression was used to examine the relationship between SES indicators (education, employment and partnership) and SGA, and whether these relationships are stable over time and different by parity. Educational attainment and employment (of the mother and her partner) were independently associated with the risk of SGA, although differences between the association between single motherhood and SGA were attenuated by adjusting for smoking status. SES differences in the risk of SGA were stable over the study period (2004-2016). The strength of these SES differences varied between mothers at their first and higher order births, with the association between maternal lower educational attainment and SGA being stronger, and the association between partner's employment and SGA being weaker, in mothers with previous live births.

Comparison with other studies

The evidence for SES inequalities by maternal educational attainment, employment and partner's employment in the risk of SGA is consistent with the literature, and the third analysis shows that these associations remain robust after limiting the sample to one record per mother. Within a systematic review of socioeconomic disparities in birth outcomes conducted in 2010 [6], 6 of the 9 (66%) studies of SGA and maternal education reported a significant association, in addition to single studies finding an association for maternal [30] and paternal employment [31]. Part of the complexity in the relationship between maternal SES and SGA results from many analyses using only one measure of SES, with maternal education [12,32] and employment [33] being the main indicators used. Factors related to the mother's partners are usually excluded, due to a lack of appropriate data or small sample sizes, despite the potential of these factors to describe the conditions mothers experience during pregnancy [34]. Whether the mother has a partner or not is largely overlooked as a risk factor in this area, with the exception of Kleijer et al (2005), who found that single mothers are at higher risk of SGA. The final estimates of SES inequalities in this study are adjusted for other SES indicators, suggesting that there are multiple pathways through which SES is linked to gestational growth.

Since the publication of Blumenshine *et al*'s systematic review [6] there has been an increased focus on how SES differences in weight outcomes at birth and during early life may be mediated through maternal BMI and smoking. In a Dutch cohort, maternal smoking and height during pregnancy were reported to explain 75% of the difference in risk of SGA between mothers with low and high education [12]. In an Australian cohort maternal smoking and the BMI of both parents were reported to explain 83.5% of SES differences in their children's BMI Z-score at age 10-11 years [36]. In the present analysis, accounting for maternal smoking reduced the magnitude of the SGA risk difference by SES from a 36% increase in risk to 20% among mothers without a university degree, and from a 48% to 31% increase in risk among unemployed mothers. Maternal smoking also explained the relatively high risk of SGA among single mothers. This attenuation corroborates previous research indicating that single mothers are more likely to smoke, and that this may be related to the level of stress that they report, relative to partnered mothers [37]. Single mothers may be relying on smoking as a means of stress relief or management during pregnancy, and smoking cessation and support programmes may be effective in reducing inequalities in birth outcomes as a result.

To our knowledge, there has been no analysis of socioeconomic inequality time trends in SGA from the mid-2000s onwards in a developed Western context. Inequalities in birth weight (adjusting for gestational age) were stable between 1961 and 2000 in a regional city-based study in North East

For peer review only - http://bmjopen.bmj.com/site/about/guidelines.xhtml

England [24], and the same is found between 2004 and 2016 in this study. The stability of SES inequalities in SGA implies that further interventions and initiatives are required to narrow SES inequalities in SGA births.

Our hypothesis was that the extent of SES inequalities in the risk of SGA may differ by parity, as the birth of the first child is a period which brings about significant physiological, lifestyle and social changes, in addition to postpartum weight retention [15]. An analysis of birth register data in Norway found that mothers who had several SGA births were characterized by low educational attainment and partners employed in low SES occupations [16]. In the present analysis, the strength of the association between SES indicators and the risk of SGA varied between primiparous and multiparous women, with education inequalities being greater for multiparous women, and employment inequalities being greater for primiparous women. The explanation may be that more advantaged women are economically able to leave the workforce after their first birth when planning further pregnancies (thus attenuating the differences between those in and outside employment when having subsequent births), whilst educational differences in terms of health behaviours, health literacy and mental wellbeing are risk factors of having repeat or new SGA outcomes [38]. This group may benefit from additional support following the birth of their first baby to promote mental and physical wellbeing and facilitate healthy behaviours.

Strengths and limitations of the study

This study benefits from a large regionally-representative sample over many years. The exposure measures are prospectively collected in the course of routine care at a regional hospital. As data from the local hospital system are used, there is no selection bias which may arise from participation in a research cohort, and the sample is therefore representative of all those receiving care under the NHS. The outcome (SGA) is derived from birth weight, which is objectively measured by a health professional at birth. The most recent birth centiles for England and Wales were used [23] to reflect changes in birth weight since the oft-used 1990 birth centiles [39]. The measures of SES used are also collected within the usual course of NHS care before birth, so the results may be used to inform risk stratification interventions at or following the booking appointment to curtail SGA births and other associated adverse health outcomes. The antenatal booking appointment is a critical point for intervention as health professionals see all mothers receiving care under the NHS. The results herein find that women who report low educational qualifications, are unemployed, or their partner is unemployed at this stage are at higher risk of SGA delivery. These groups, as well as women with no partners and/or other social support at the time of the booking appointment, may then be referred for additional support to minimize the risk of an SGA birth and other adverse maternal and health outcomes. A limitation of our dataset is that such processes (if they y) were not electronically recorded and hence not includes in our analyses.

Some potential risk factors were not adjusted for in this study due to inconsistency of data for those specific variables as captured routinely in antenatal care, including diet during pregnancy and alcohol intake. These factors may also be mediate the effect of SES on SGA risk, wherein disadvantaged SES groups could be more likely to engage in risky health behaviours. In addition, this analysis did not account for characteristics of the residential environments mothers lived in during pregnancy. Systematic reviews indicate that social, built and air characteristics of the environment experienced during pregnancy are strongly associated with birth outcomes [5,40], and this will be addressed in a follow-up study on the associations between environmental characteristics and birth outcomes for the cohort.

For peer review only - http://bmjopen.bmj.com/site/about/guidelines.xhtml

As the data used in this study are limited to a hospital serving the city of Southampton and the surrounding region, the results may not apply to hospitals serving populations with differing characteristics. Southampton is a provincial urban city which is more deprived than the average Local Authority in England, although the surrounding area (Hampshire) is relatively affluent [41]. Southampton has a similar ethnicity profile to the rest of England and Wales [42], but with a relatively large university student population, and women in Southampton are underrepresented in managerial, administrative and professional occupations, relative to others in England [43]. As a result, findings from this study may not be replicated using healthcare records in areas with predominantly rural populations, or areas with non-student and managerial populations.

Implications for research and practice

The persistence of educational and employment inequalities in the risk of SGA found within this study justifies further interventions and initiatives in order to narrow SES inequalities in the risk of SGA, and subsequently their long-term adverse health impact. The antenatal booking appointment offers an opportune moment for risk stratification and signposting of additional support for women with low educational qualification, in unemployed households and low social support. Smoking appeared as a potential mediator for SES inequalities in this study, despite support in smoking cessation being offered in the course of NHS care [44]. This suggests that further support is required for mothers of low SES, and pre- and interconception programmes may have the added benefit of reducing the extent of SES inequalities in SGA, in addition to overall SGA rates. For research, this study aligns with recent calls to incorporate paternal/partner influences in developmental health research [34], in that similar levels of SGA risk are found for maternal and partner unemployment. Research in this area should adopt a more family-centred approach in relation to offspring health outcomes, taking into account contributing exposures from others within the household structure (partners and siblings).

Conclusions

This study confirms that socioeconomic status indicators, including educational attainment, employment status and single motherhood, are strongly and independently associated with the risk of small for gestational age birth, and they are not narrowing over time. Maternal smoking appears to play a significant role in these inequalities, particularly for lone mothers. However, the associations between educational attainment and employment status with SGA risk remain strong even after accounting for maternal smoking and BMI. Inequalities in SGA risk by maternal educational attainment appear greater for multiparous compared to primiparous women, while the opposite is true by maternal employment status. Further research is needed to identify critical windows of opportunity (preconception/pregnancy/interconception) and effective interventions in order to narrow these inequalities. Prevention programmes targeting socioeconomically disadvantaged women which incorporate smoking cessation and social support are vital to tackling health inequalities in SGA.

For peer review only - http://bmjopen.bmj.com/site/about/guidelines.xhtml

What is already known on this topic

- Babies born to mothers in low socioeconomic status (SES) are at higher risk of being born small for gestational age (SGA).

- These SES inequalities were found to be stable between 1961 and 2000 in a previous English study.

- The relationship between maternal SES and SGA is linked to a higher prevalence of smoking and maternal underweight among mothers in low SES.

What this study adds

- Indicators of parental SES (maternal education, maternal and partner employment) are independently associated with the risk of being born SGA with some variation in the magnitude of risk between primiparous and multiparous women, while the risk difference between lone and partnered mothers is attenuated by accounting for maternal smoking.

-These SES inequalities remained stable between 2004 and 2016 in this English population-based cohort.

For peer review only

Footnotes

Contributors

NAA is the Principal Investigator of the project, and acts as the guarantor of this study. SW, NZ, PR, DS, DC, NMc, MH and NAA contributed to study conception and design. NMc provided input on the statistical analysis for this study. SW conducted the statistical analyses, and drafted the initial report. NZ checked the accuracy of the reported estimates from the statistical models. All authors contributed to interpretation of data and revising the manuscript critically for important intellectual content. All authors read and approved the final manuscript.

Funding

This research is supported by an Academy of Medical Sciences and Wellcome Trust grant to NAA (Grant no: AMS_HOP001\1060), and the National Institute for Health Research through the NIHR Southampton Biomedical Research Centre. The research funders had no input on research design or manuscript drafting. MAH is supported by the British Heart Foundation and the National Institute for Health Research through the NIHR Southampton Biomedical Research Centre.

Acknowledgments

We thank Ravita Taheem (Senior Public Health Practitioner, Southampton City Council) for her input in the conception of this paper, David Cable (Electronic Patient Records Implementation and Service Manager) and Florina Borca (Senior Information Analyst for R&D, NIHR Southampton Biomedical Research Centre) at University Hospital Southampton NHS Foundation Trust for their support in extracting the data used in this study.

Competing interests

All authors have completed the ICMJE uniform disclosure form at www.icmje.org/coi_disclosure.pdf and declare: NAA had financial support from the Academy of Medical Sciences/Wellcome Trust and the NIHR Southampton Biomedical Research Centre for the submitted work; no financial relationships with any organisations that might have an interest in the submitted work in the previous three years; no other relationships or activities that could appear to have influenced the submitted work.

Ethical approval

This study used anonymised antenatal record data supplied by University Hospital Southampton Trust. This analysis forms part of a research project reviewed and approved by the University of Southampton Faculty of Medicine Ethics Committee (ref 24433) and the National Health Service Health Research Authority (ref 242031).

Data sharing

The authors' ethical approval from the Faculty of Medicine Ethics Committee, University of Southampton (Reference number 24433) restricts public sharing of the data used in this study. The data owner is University Hospital Southampton NHS Trust. Please contact NAA to request data access beyond that included in the manuscript. Further ethical and research governance approval may be required.

Transparency

The first author, SW, affirms that the manuscript is an honest, accurate, and transparent account of the study being reported.

For peer review only - http://bmjopen.bmj.com/site/about/guidelines.xhtml

References

1 Mendez-Figueroa H, Truong V, Pedroza C, *et al.* Morbidity and mortality with small for gestational age: Secondary analysis of nine MFMU network studies. Am. J. Obstet. Gynecol. 2016;**214**:S320–1.http://ovidsp.ovid.com/ovidweb.cgi?T=JS&PAGE=reference&D=emed13&NEWS=N&AN=72164789

2 Reilly JJ, Armstrong J, Dorosty AR, *et al.* Early life risk factors for obesity in childhood: cohort study. *BMJ* 2005;**330**:1357.http://www.bmj.com/content/330/7504/1357.abstract

3 McCowan L, Horgan RP. Risk factors for small for gestational age infants. *Best Pract Res Clin Obstet Gynaecol* 2009;**23**:779–93. doi:https://doi.org/10.1016/j.bpobgyn.2009.06.003

4 Mitchell EA, Robinson E, Clark PM, *et al.* Maternal nutritional risk factors for small for gestational age babies in a developed country: a case-control study. *Arch Dis Child - Fetal Neonatal Ed* 2004;**89**:F431 LP-F435.http://fn.bmj.com/content/89/5/F431.abstract

5 Vos AA, Posthumus AG, Bonsel GJ, *et al.* Deprived neighborhoods and adverse perinatal outcome: a systematic review and meta-analysis. *Acta Obstet Gynecol Scand* 2014;**93**:727–40. doi:10.1111/aogs.12430

6 Blumenshine P, Egerter S, Barclay CJ, *et al.* Socioeconomic Disparities in Adverse Birth Outcomes: A Systematic Review. *Am J Prev Med* 2010;**39**:263–72. doi:https://doi.org/10.1016/j.amepre.2010.05.012

7 Gao W, Paterson J, Carter S, *et al.* Risk factors for preterm and small-for-gestational-age babies: a cohort from the Pacific Islands Families Study. *J Paediatr Child Health* 2006;**42**:785–92. doi:10.1111/j.1440-1754.2006.00978.x

8 Luo Z-C, Wilkins R, Kramer MS, *et al.* Effect of neighbourhood income and maternal education on birth outcomes: a population-based study. *C Can Med Assoc J* 2006;**174**:1415–20. doi:10.1503/cmaj.051096

9 Masi CM, Hawkley LC, Piotrowski ZH, *et al.* Neighborhood economic disadvantage, violent crime, group density, and pregnancy outcomes in a diverse, urban population. *Soc Sci Med* 2007;**65**:2440–57. doi:10.1016/j.socscimed.2007.07.014

10 Pevalin DJ, Wade TJ, Brannigan A, *et al.* Beyond biology: The social context of prenatal behaviour and birth outcomes. *Soz Praventivmed* 2001;**46**:233–9. doi:10.1007/BF01593178

11 Savitz DA, Kaufman JS, Dole N, *et al.* Poverty, education, race, and pregnancy outcome. *Ethn Dis* 2004;**14**:322–9.

12 van den Berg G, van Eijsden M, Galindo-Garre F, *et al.* Smoking overrules many other risk factors for small for gestational age birth in less educated mothers. *Early Hum Dev* 2013;**89**:497–501. doi:https://doi.org/10.1016/j.earlhumdev.2013.03.007

13 Love C, David RJ, Rankin KM, *et al.* Exploring Weathering: Effects of Lifelong Economic Environment and Maternal Age on Low Birth Weight, Small for Gestational Age, and Preterm Birth in African-American and White Women. *Am J Epidemiol* 2010;**172**:127–34.http://dx.doi.org/10.1093/aje/kwq109

14 Taylor-Robinson D, Agarwal U, Diggle PJ, *et al.* Quantifying the Impact of Deprivation on Preterm Births: A Retrospective Cohort Study. *PLoS One* 2011;**6**:e23163.https://doi.org/10.1371/journal.pone.0023163

15 Hollis JL, Crozier SR, Inskip HM, *et al.* Modifiable risk factors of maternal postpartum weight

For peer review only - http://bmjopen.bmj.com/site/about/guidelines.xhtml

retention: An analysis of their combined impact and potential opportunities for prevention. *Int J Obes* 2017;**41**:1091–8. doi:10.1038/ijo.2017.78

16 Bakketeig LS, Bjerkedal T, Hoffman HJ. Small-for-gestational age births in successive pregnancy outcomes: results from a longitudinal study of births in Norway. *Early Hum Dev* 1986;**14**:187–200. doi:https://doi.org/10.1016/0378-3782(86)90180-5

17 Milgrom J, Gemmill AW, Bilszta JL, *et al.* Antenatal risk factors for postnatal depression: A large prospective study. *J Affect Disord* 2008;**108**:147–57. doi:https://doi.org/10.1016/j.jad.2007.10.014

18 Patel V, Rodrigues M, DeSouza N. Gender, poverty, and postnatal depression: A study of mothers in Goa, India. *Am J Psychiatry* Published Online First: 2002. doi:10.1176/appi.ajp.159.1.43

19 Richard B, Monica CD, Costas M, *et al.* Female Labor Supply, Human Capital, and Welfare Reform. *Econometrica* 2016;**84**:1705–53. doi:10.3982/ECTA11576

20 Rafferty A, Wiggan J. The Time-related Underemployment of Lone Parents during Welfare Reform, Recession and Austerity: A Challenge to In-work Conditionality? *Soc Policy Adm* 2017;**51**:511–38. doi:10.1111/spol.12190

21 Jofre-Bonet M, Serra-Sastre V, Vandoros S. The impact of the Great Recession on health-related risk factors, behaviour and outcomes in England. *Soc Sci Med* 2018;**197**:213–25. doi:https://doi.org/10.1016/j.socscimed.2017.12.010

22 National Institute for Clinical Excellence. Antenatal care for uncomplicated pregnancies: schedule of appointments. 2012.https://pathways.nice.org.uk/pathways/antenatal-care-for-uncomplicated-pregnancies/antenatal-care-for-uncomplicated-pregnancies-schedule-of-appointments.pdf (accessed 23 Jul 2018).

23 Norris T, Seaton SE, Manktelow BN, *et al.* Updated birth weight centiles for England and Wales. *Arch Dis Child - Fetal Neonatal Ed* Published Online First: 7 December 2017.http://fn.bmj.com/content/early/2017/12/07/archdischild-2017-313452.abstract

24 Glinianaia S V, Ghosh R, Rankin J, *et al.* No improvement in socioeconomic inequalities in birthweight and preterm birth over four decades: a population-based cohort study. *BMC Public Health* 2013;**13**:345. doi:10.1186/1471-2458-13-345

25 Kozuki N, Lee ACC, Silveira MF, *et al.* The associations of parity and maternal age with small-for-gestational-age, preterm, and neonatal and infant mortality: a meta-analysis. *BMC Public Health* 2013;**13**:S2–S2. doi:10.1186/1471-2458-13-S3-S2

26 Block-Abraham DM, Adamovich D, Turan OM, *et al.* Maternal blood pressures during pregnancy and the risk of delivering a small-for-gestational-age neonate. *Hypertens pregnancy* 2016;**35**:350–60. doi:10.3109/10641955.2016.1150487

27 Ranganathan P, Pramesh CS, Buyse M. Common pitfalls in statistical analysis: The perils of multiple testing. *Perspect Clin Res* 2016;**7**:106–7. doi:10.4103/2229-3485.179436

28 Baron RM, Kenny DA. The moderator-mediator variable distinction in social psychological research: conceptual, strategic, and statistical considerations. *J Pers Soc Psychol* 1986;**51**:1173–82.

29 Galobardes B, Costanza MC, Bernstein MS, *et al.* Trends in Risk Factors for Lifestyle-Related Diseases by Socioeconomic Position in Geneva, Switzerland, 1993–2000: Health Inequalities Persist. *Am J Public Health* 2003;**93**:1302–9. doi:10.2105/AJPH.93.8.1302

For peer review only - http://bmjopen.bmj.com/site/about/guidelines.xhtml

30 Gissler M, Rahkonen O, Arntzen A, *et al.* Trends in socioeconomic differences in Finnish perinatal health 1991–2006. *J Epidemiol Community Health* 2009;**63**:420 LP-425.http://jech.bmj.com/content/63/6/420.abstract

31 Fairley L, Leyland AH. Social class inequalities in perinatal outcomes: Scotland 1980–2000. *J Epidemiol Community Health* 2006;**60**:31 LP-36.http://jech.bmj.com/content/60/1/31.abstract

32 Agyemang C, Vrijkotte TGM, Droomers M, *et al.* The effect of neighbourhood income and deprivation on pregnancy outcomes in Amsterdam, The Netherlands. *J Epidemiol Community Health* 2009;**63**:755 LP-760.http://jech.bmj.com/content/63/9/755.abstract

33 Räisänen S, Gissler M, Sankilampi U, *et al.* Contribution of socioeconomic status to the risk of small for gestational age infants – a population-based study of 1,390,165 singleton live births in Finland. *Int J Equity Health* 2013;**12**:28. doi:10.1186/1475-9276-12-28

34 Sharp GC, Lawlor DA, Richardson SS. It's the mother!: How assumptions about the causal primacy of maternal effects influence research on the developmental origins of health and disease. *Soc Sci Med* 2018;**213**:20–7. doi:https://doi.org/10.1016/j.socscimed.2018.07.035

35 Kleijer ME, Dekker GA, Heard AR. Risk factors for intrauterine growth restriction in a socio-economically disadvantaged region. *J Matern Neonatal Med* 2005;**18**:23–30. doi:10.1080/14767050500127674

36 Iguacel I, Chung A, Gearon E, *et al.* Influence of early-life risk factors on socioeconomic inequalities in weight gain. *J Public Health (Bangkok)* Published Online First: 26 March 2018.http://dx.doi.org/10.1093/pubmed/fdy056

37 Sperlich S, Maina MN, Noeres D. The effect of psychosocial stress on single mothers' smoking. *BMC Public Health* 2013;**13**:1125. doi:10.1186/1471-2458-13-1125

38 Güneş PM. The role of maternal education in child health: Evidence from a compulsory schooling law. *Econ Educ Rev* 2015;**47**:1–16. doi:https://doi.org/10.1016/j.econedurev.2015.02.008

39 J. CT, V. FJ, A. PM. British 1990 growth reference centiles for weight, height, body mass index and head circumference fitted by maximum penalized likelihood. *Stat Med* 1998;**17**:407–29. doi:10.1002/(SICI)1097-0258(19980228)17:4<407::AID-SIM742>3.0.CO;2-L

40 X. L, S. H, A. J, *et al.* Association between ambient fine particulate matter and preterm birth or term low birth weight: An updated systematic review and meta-analysis. *Environ Pollut* 2017;**227**:596–605. doi:http://dx.doi.org/10.1016/j.envpol.2017.03.055

41 Department for Communities and Local Government. English Indices of Deprivation 2015. 2015.https://www.gov.uk/government/uploads/system/uploads/attachment_data/file/467764/File_1_ID_2015_Index_of_Multiple_Deprivation.xlsx (accessed 11 Sep 2018).

42 Office for National Statistics. Ethnicity and National Identity in England and Wales 2011. 2012.https://www.ons.gov.uk/peoplepopulationandcommunity/culturalidentity/ethnicity/articles/ethnicityandnationalidentityinenglandandwales/2012-12-11 (accessed 11 Sep 2018).

43 Office for National Statistics. 2011 Census: Key statistics and quick statistics for local authorities in the United Kingdom - part 2. 2014.https://www.ons.gov.uk/employmentandlabourmarket/peopleinwork/employmentandemployeetypes/datasets/2011censuskeystatisticsandquickstatisticsforlocalauthoritiesintheunitedkingdompart2 (accessed 13 Feb 2018).

For peer review only - http://bmjopen.bmj.com/site/about/guidelines.xhtml

44 National Institute for Clinical Excellence. Smoking: stopping in pregnancy and after childbirth. 2010.https://www.nice.org.uk/guidance/ph26 (accessed 11 Sep 2018).

For peer review only

For peer review only - http://bmjopen.bmj.com/site/about/guidelines.xhtml

STROBE Statement—checklist of items that should be included in reports of observational studies

	Item No	Recommendation	Page No
**Title and abstract**	1	(*a*) Indicate the study's design with a commonly used term in the title or the abstract	1
		(*b*) Provide in the abstract an informative and balanced summary of what was done and what was found	2
**Introduction**			
Background/rationale	2	Explain the scientific background and rationale for the investigation being reported	3
Objectives	3	State specific objectives, including any prespecified hypotheses	3
**Methods**			
Study design	4	Present key elements of study design early in the paper	3
Setting	5	Describe the setting, locations, and relevant dates, including periods of recruitment, exposure, follow-up, and data collection	4
Participants	6	(*a*) *Cohort study*—Give the eligibility criteria, and the sources and methods of selection of participants. Describe methods of follow-up<br>*Case-control study*—Give the eligibility criteria, and the sources and methods of case ascertainment and control selection. Give the rationale for the choice of cases and controls<br>*Cross-sectional study*—Give the eligibility criteria, and the sources and methods of selection of participants	4
		(*b*) *Cohort study*—For matched studies, give matching criteria and number of exposed and unexposed<br>*Case-control study*—For matched studies, give matching criteria and the number of controls per case	N/A
Variables	7	Clearly define all outcomes, exposures, predictors, potential confounders, and effect modifiers. Give diagnostic criteria, if applicable	4-5
Data sources/ measurement	8*	For each variable of interest, give sources of data and details of methods of assessment (measurement). Describe comparability of assessment methods if there is more than one group	4-5
Bias	9	Describe any efforts to address potential sources of bias	4
Study size	10	Explain how the study size was arrived at	4
Quantitative variables	11	Explain how quantitative variables were handled in the analyses. If applicable, describe which groupings were chosen and why	5-6
Statistical methods	12	(*a*) Describe all statistical methods, including those used to control for confounding	5
		(*b*) Describe any methods used to examine subgroups and interactions	5
		(*c*) Explain how missing data were addressed	5
		(*d*) *Cohort study*—If applicable, explain how loss to follow-up was addressed<br>*Case-control study*—If applicable, explain how matching of cases and controls was addressed<br>*Cross-sectional study*—If applicable, describe analytical methods taking account of sampling strategy	N/A
		(*e*) Describe any sensitivity analyses	5

Continued on next page

For peer review only - http://bmjopen.bmj.com/site/about/guidelines.xhtml

**Results**			
Participants	13*	(a) Report numbers of individuals at each stage of study—eg numbers potentially eligible, examined for eligibility, confirmed eligible, included in the study, completing follow-up, and analysed	4-6
		(b) Give reasons for non-participation at each stage	N/A
		(c) Consider use of a flow diagram	N/A
Descriptive data	14*	(a) Give characteristics of study participants (eg demographic, clinical, social) and information on exposures and potential confounders	4-7
		(b) Indicate number of participants with missing data for each variable of interest	6-7
		(c) *Cohort study*—Summarise follow-up time (eg, average and total amount)	N/A
Outcome data	15*	*Cohort study*—Report numbers of outcome events or summary measures over time	N/A
		Case-control study—Report numbers in each exposure category, or summary measures of exposure	N/A
		Cross-sectional study—Report numbers of outcome events or summary measures	6-7
Main results	16	(*a*) Give unadjusted estimates and, if applicable, confounder-adjusted estimates and their precision (eg, 95% confidence interval). Make clear which confounders were adjusted for and why they were included	8,10,14,15
		(*b*) Report category boundaries when continuous variables were categorized	6-7
		(*c*) If relevant, consider translating estimates of relative risk into absolute risk for a meaningful time period	N/A
Other analyses	17	Report other analyses done—eg analyses of subgroups and interactions, and sensitivity analyses	14-15
**Discussion**			
Key results	18	Summarise key results with reference to study objectives	16
Limitations	19	Discuss limitations of the study, taking into account sources of potential bias or imprecision. Discuss both direction and magnitude of any potential bias	17-18
Interpretation	20	Give a cautious overall interpretation of results considering objectives, limitations, multiplicity of analyses, results from similar studies, and other relevant evidence	18
Generalisability	21	Discuss the generalisability (external validity) of the study results	18
**Other information**			
Funding	22	Give the source of funding and the role of the funders for the present study and, if applicable, for the original study on which the present article is based	20

*Give information separately for cases and controls in case-control studies and, if applicable, for exposed and unexposed groups in cohort and cross-sectional studies.

Note: An Explanation and Elaboration article discusses each checklist item and gives methodological background and published examples of transparent reporting. The STROBE checklist is best used in conjunction with this article (freely available on the Web sites of PLoS Medicine at http://www.plosmedicine.org/, Annals of Internal Medicine at http://www.annals.org/, and Epidemiology at http://www.epidem.com/). Information on the STROBE Initiative is available at www.strobe-statement.org.

For peer review only

BMJ Open

Are socioeconomic inequalities in the incidence of small-for-gestational-age birth narrowing? Findings from a population-based cohort in the South of England

Journal:	*BMJ Open*
Manuscript ID	bmjopen-2018-026998.R1
Article Type:	Research
Date Submitted by the Author:	12-Jun-2019
Complete List of Authors:	Wilding, Sam; University of Southampton, Academic Unit of Primary Care and Population Sciences, Faculty of Medicine<br>Ziauddeen, Nida; University of Southampton, Academic Unit of Primary Care and Population Sciences, Faculty of Medicine<br>Roderick, Paul; University of Southampton, Academic Unit of Primary Care and Population Sciences, Faculty of Medicine<br>Smith, Dianna; University of Southampton, Geography & Environment<br>Chase, Debbie; Southampton City Council, Public Health<br>Macklon, Nick; University of Copenhagen, Department of Obstetrics and Gynaecology; London Women's Clinic<br>McGrath, Nuala; University of Southampton, Academic Unit of Primary Care and Population Sciences, Faculty of Medicine; University of Southampton, School of Economic, Social and Political Science, Faculty of Social Sciences,<br>Hanson, Mark; University of Southampton, Institute of Developmental Sciences, Academic Unit of Human Development and Health, Faculty of Medicine; University of Southampton and University Hospital Southampton NHS Foundation Trust , NIHR Southampton Biomedical Research Centre<br>Alwan, Nisreen; University of Southampton, Academic Unit of Primary Care and Population Sciences, Faculty of Medicine; University of Southampton and University Hospital Southampton NHS Foundation Trust, NIHR Southampton Biomedical Research Centre
**Primary Subject Heading**:	Public health
Secondary Subject Heading:	Paediatrics, Obstetrics and gynaecology
Keywords:	PUBLIC HEALTH, SOCIAL MEDICINE, small for gestational age, socioeconomic inequalities

SCHOLARONE™ Manuscripts

Are socioeconomic inequalities in the incidence of small-for-gestational-age birth narrowing? Findings from a population-based cohort in the South of England

Authors:

Sam Wilding[1], Nida Ziauddeen[1], Paul Roderick[1], Dianna Smith[2], Debbie Chase[3], Nicholas Macklon[4,5], Nuala McGrath[1,6], Mark Hanson[7,8], Nisreen A Alwan[1,8]

Author affiliations:

[1] School of Primary Care and Population Sciences, Faculty of Medicine, University of Southampton

[2] Geography and Environmental Science, Faculty of Environmental and Life Sciences, University of Southampton

[3] Public Health, Southampton City Council

[4] Department of Obstetrics and Gynaecology, University of Copenhagen, Zealand University Hospital, Roskilde 4000, Denmark

[5] London Women's Clinic, 113-115 Harley Street, London W1G 6AP

[6] School of Economic, Social and Political Science, Faculty of Social Sciences, University of Southampton

[7] Institute of Developmental Sciences, School of Human Development and Health, Faculty of Medicine, University of Southampton

[8] NIHR Southampton Biomedical Research Centre, University of Southampton and University Hospital Southampton NHS Foundation Trust

Corresponding author:

Nisreen A Alwan, Room AC23, South Academic Block, University Hospital Southampton, Southampton, UK, SO16 6YD. Email: n.a.alwan@soton.ac.uk.

For peer review only - http://bmjopen.bmj.com/site/about/guidelines.xhtml

Abstract

Objectives

To investigate socioeconomic inequalities, using maternal educational attainment, maternal and partner employment status, and lone motherhood indicators, in the risk of small for gestational age (SGA) births, their time trend, potential mediation by maternal smoking and body mass index (BMI), and effect modification by parity.

Design

Population-based birth cohort utilising routine antenatal healthcare data.

Setting

Babies born at University Hospital Southampton, UK, between 2004 and 2016.

Participants

65,909 singleton live births born to mothers aged ≥18 years between 24 and 42 weeks gestation.

Main outcome measures

SGA (birth weight <10th percentile for others born at the same number of completed weeks compared to 2013/2014 within England and Wales).

Results

Babies born to mothers educated up to secondary school level (adjusted Odds Ratio (aOR) 1.30, 99% Confidence Interval (CI) 1.17-1.45), who were unemployed (aOR 1.27, CI 1.16-1.38) or with unemployed partners (aOR 1.27, CI 1.13-1.43) were at greater risk of being SGA. There was no statistically significant change in the magnitude of this risk difference by these indicators over time between 2004 and 2016, as estimated by linear interactions with year of birth. Babies born to lone mothers were not at higher risk compared to partnered mothers after adjusting for maternal smoking (aOR 1.06, CI 0.93-1.20). The inverse association between maternal educational attainment and SGA risk appeared greater in multiparous (aOR 1.38, CI 1.09-1.75) compared to primiparous women (aOR 1.26, CI 1.11-1.44), and the reverse was true for maternal and partner's unemployment where the association was stronger in primiparous women.

Conclusions

Socioeconomic inequalities in SGA risk by educational attainment and employment status are not narrowing over time, with differences in association strength by parity. The greater SGA risk in lone mothers was potentially explained by maternal smoking. Preventive interventions should target socially disadvantaged women, including preconception and postpartum smoking cessation to reduce SGA risk.

Strengths and limitations of this study

- This study uses a relatively-large sample of population-level antenatal care data to predict the risk of small-for-gestational-age births by socioeconomic factors
- Standard routinely-collected measures recorded at the first antenatal appointment are utilised which can be used for risk prediction in practice without the need to collect extra data during antenatal appointments

For peer review only

For peer review only - http://bmjopen.bmj.com/site/about/guidelines.xhtml

- Limitations include the transferability of results from this population to others with differing characteristics, that socioeconomic factors were only assessed at one time point in pregnancy, and self-reporting of educational qualifications and employment.

For peer review only

For peer review only - http://bmjopen.bmj.com/site/about/guidelines.xhtml

Introduction

Babies born small for gestational age (SGA) are at higher risk of neonatal morbidity, mortality [1] and childhood obesity potentially through compensatory early growth [2][3]. Numerous clinical and lifestyle risk factors are associated with the risk of being SGA, including maternal height, weight, diet, ethnicity, parity, smoking, pre-eclampsia and hypertension [4,5]. Closely linked to these risk factors there is extensive evidence of socioeconomic status (SES) inequalities, with more SGA babies born to mothers living in the most deprived communities compared to those in the most affluent [6].

Several proxies of SES are present in the literature, with area measures of wealth, maternal education, employment and income being the most common indicators, while paternal factors being notably absent [7]. Disadvantaged SES groups (in terms of education and income) typically experience greater rates of SGA births [8,9]. The majority of studies rely on one proxy of SES, but studies controlling for several SES measures find that different aspects of SES are independently associated with the risk of SGA [10–14].

Despite the wealth of research on the association between parental SES and SGA, the underlying mechanisms are poorly understood [15]. Current explanations focus on the availability of (physiological and material) resources and mediating factors that differ between women of high and low SES. For resources, the 'weathering' hypothesis states that women in low SES at the time of conception have experienced relatively high levels of cumulative disadvantage in terms of income, stress and diet, which have led to a deterioration in physiological health [16]. This association may also be mediated by lifestyle factors, wherein mothers in low SES are more likely to be exposed to or partake in risk factors for SGA such as smoking. Mediation analyses have found that higher rates of underweight and smoking at conception among mothers with low educational attainment mediates the association between SES and birth outcomes in the UK [15,17].

The extent of these SES inequalities in the risk of SGA may differ between first and higher order births. The birth of the first child brings significant physiological, wellbeing and social changes [18], and women in low SES may have weaker social support mechanisms to adjust to these changes, as they appear to be at higher risk of SGA in subsequent births after adjusting for clinical risk factors [19]. Risk factors for SGA specific to second and higher order births are more prevalent in women of low SES, with postnatal depression being more common in mothers without a university degree and those in poverty [20,21].

In England, public health policy aims to narrow SES inequalities in birth outcomes over time [22,23], and changes in the extent of inequalities in SGA have been noted in other European countries since the early-2000s [24]. Major welfare reforms enacted in the UK between 1999 and 2002 increased in-work tax incentives, which particularly increased the net income of part-time working women, relative to those out of work [25]. In 2008 the global 'great recession' occurred, after which single mothers in England became increasingly less likely to be employed, whilst facing disproportionate losses of welfare income, facing a double income penalty relative to working mothers [26]. The recession appears to have had differential impacts on women by level of educational attainment, with those without a university degree experiencing a post-recession rise in the prevalence of obesity, relative to those with degrees [27].

Utilising an antenatal healthcare database in Hampshire, England, we aimed to examine differences in SGA risk by SES indicators, investigate if these differences are mediated by maternal body mass index and smoking, and whether the inequalities gap has narrowed over the 13 year study period

For peer review only - http://bmjopen.bmj.com/site/about/guidelines.xhtml

(2004-2016). In addition, we aimed to stratify by parity in order to examine whether the SES gap in SGA risk is the same at first births, relative to 2nd and higher order births.

Methods

Data

This analysis is based on a population-based cohort including anonymised antenatal and delivery records of women aged ≥18 years who had a live singleton birth between 1 January 2004 and 31 December 2016 at the University Hospital Southampton (UHS) National Health Service (NHS) Trust in the South of England. UHS is the primary centre for maternity care for the city of Southampton and the surrounding areas, and is the regional centre for high-risk pregnancies. The process of deriving a sample for analysis is outlined in Supplementary Figure 1. To ensure that the findings are applicable to the majority of (non high-risk) pregnancies, records with late first antenatal (booking) appointments (after 24 weeks gestation, as assessed by ultrasound) and of mothers under the age of 18 were excluded. First, we analysed the risk of SGA by SES in all births (including more than one birth per mother if in the database and study timeframe), adjusting for confounding and clustering. We then tested whether differences between SES groups (by maternal education, employment, paternal employment and partnership status) have changed over the study period (2004-2016). We then limited the analysis to the first recorded birth per mother in the dataset, and stratified by parity (primiparous and multiparous), to avoid biasing sub-analyses via double-counting. This project was approved by the University of Southampton Faculty of Medicine Ethics Committee (ref 24433) and the NHS Health Research Authority (ref 242031).

Assessment of SES exposures

Socioeconomic measures were self-reported at the first antenatal (booking) appointment, which is recommended by the National Institute for Health and Care Excellence (NICE) Antenatal Care Guidelines to occur by the 10th week of gestation [28]. Mothers were asked to report their highest educational qualification, classified as university degree (highest level), college (A levels) or secondary school (GCSE), whether they were currently employed, and if their partners were currently employed (possible answers included employed, unemployed and seeking work or student, with the latter two being combined). Partnership status was self-reported at the same appointment. All four SES proxies were categorised to be compared to mothers with advantaged SES (mother has a university degree; mother is employed; mother's partner is employed; mother has a partner). Time trends in SES factors were examined, and presented in Supplementary Figure 2.

Assessment of outcome

Birth weight was measured by healthcare professionals for all births in the dataset. Gestational age was based on a dating ultrasound scan performed by healthcare professionals, and was present for all records in the dataset. Birth weight centile for gestational age is calculated using reference values provided in the most recently released data (2013-2014) for England and Wales, which were validated using 2015 records [29]. Given that the association between SES and preterm births is well established in the literature [30], and that gestational age is strongly associated with birthweight, we use a Small for Gestational Age (SGA) measure to assess low birth weight rather than the standard birth weight cut-off.

The birth centile references are available for 24-42 completed weeks of gestation, so live births at ≤23 (71) or >42 (568) completed weeks or with indeterminate sex (16) are excluded from the analysis (SGA sample = 65,909/66,564). In line with World Health Organisation guidelines, UK

For peer review only - http://bmjopen.bmj.com/site/about/guidelines.xhtml

guidelines and common practice, SGA is defined as a birth weight lower than the 10th percentile compared to others born at the same number of weeks gestation in the sex-specific reference centiles [31–33], and all others are defined as Not Small for Gestational Age (non-SGA).

Assessment of confounder and mediator variables

Maternal age, weight, height, parity, ethnicity and smoking history were self-reported at the booking antenatal appointment. Baby's sex was assessed at birth by a healthcare professional. Maternal weight and blood pressure were measured by a healthcare professional at the booking appointment, and screening for gestational diabetes was carried out for women identified as at high risk in the second trimester of pregnancy [28]. Maternal age, ethnicity, gestational diabetes and systolic blood pressure were adjusted for in the multivariable models, as these factors have been associated with size at birth in previous analyses [4,34,35]. Parity (no versus 1 or more previous births) was treated as a confounder in the models analysing the whole sample, and then as an effect modifier for SES through interaction terms and later stratification. Maternal body mass index (BMI) and smoking history are included as potential mediators of the relationship between SES and risk of SGA, based on previous evidence [15,17]. Maternal BMI was categorised as underweight (<18.5), normal (18.5-24.9), overweight/pre-obese (25.0-29.9) and obese (30+) [36], and treated as a categorical variable in all analyses. Maternal smoking was reported as follows (never smoked, ex-smoker, <10 per day, 10-20 per day and 20+ per day), and also treated as a categorical variable in all analyses.

Patient and Public Involvement

Because this analysis uses routinely-collected antenatal data where patient identifiers were anonymised, no patients or members of the public were recruited or consulted by the research team.

Statistical analysis

All analyses were conducted using Stata 15 (College Station, Texas). Descriptive statistics and the unadjusted odds ratios (ORs) between all variables and risk of SGA are presented in Table 1. T-tests were used to test whether the mean of each continuous variable (maternal BMI, age and systolic blood pressure at booking) differed between those born SGA and non-SGA. Multivariable logistic regression models were used to estimate ORs, p-values and respective 99% confidence intervals (CI) for SES differences in the risk of SGA independently after adjustment for control variables, after adjustment for other SES indicators, and then after controlling for mediators. A p-value cut-off of 0.01 is used to test for statistical significance when reporting risk rather than the more conventional 0.05 cut-off in order to minimise the risk of type I error due to multiple testing, as adjusted models control for multiple SES indicators [37]. Evidence of mediation is examined through assessing the attenuation of SES with SGA associations once known risk factors are controlled for, and the significance once each a priori mediator (first BMI, then smoking) is controlled for [38]. In all logistic regressions, cases with missing data for variables within the model were dropped (complete case analysis).

In the first analysis, adjusted ORs for the risk of a baby being born SGA are presented in model 1 (control variables include maternal age, parity, ethnicity, gestational diabetes, gestational hypertension and systolic blood pressure at booking) independently for maternal education, employment and partnership status, adjusting for clustering of births within the same mother. In model 2, all three of these SES proxies are controlled for, in additional to the control variables in model 1, before including the two mediators (maternal BMI and smoking) sequentially in models 3

For peer review only - http://bmjopen.bmj.com/site/about/guidelines.xhtml

and 4. Due to collinearity between maternal partnership and partner's employment, the association for the latter is tested separately with the same structure.

In the second analysis, year and the interactions between year and SES indicator (slope) effects are included to model 4 for maternal education, employment, partner's employment and partnership status, to test whether SES inequalities in the risk of being born SGA are widening or narrowing over time during the study period. These slopes represent the change in relative odds of SGA for the socioeconomic group relative to the control group for each year in the dataset (2004-2016). Odds ratios >1 indicate that this group became at higher risk of SGA births over time, relative to the control group [39]. Further models were estimated including SES interactions between a dummy indicator for records pre- (2004-2008) and post- (2009-2016) 2008, to test whether SES inequalities in the risk of SGA changed in magnitude between the two periods.

In the third analysis, the sample is limited to the first birth for each mother (1 birth per mother), and then stratified by parity (primiparous or multiparous). Limiting the sample to the first birth for each mother acts as a sensitivity analysis for the first analysis, ensuring that the results are not influenced by multiple births per mother. Interactions between SES and parity are estimated to test whether the association between SES and risk of SGA is modified by parity, and then parity-stratified modelling was conducted. A p-value cut-off of 0.05 is used to test for interactions. As in the first analysis, adjusted SES ORs are presented for each sub-sample, then these ORs are adjusted for other SES indicators, before including mediators (maternal BMI and smoking).

For peer review only - http://bmjopen.bmj.com/site/about/guidelines.xhtml

Results

There are 65,825 singleton live births within the dataset which can be categorised as SGA or non-SGA to 44,371 mothers. Of births, 71% were to women with no university degree, in employment (67.9%), have partners the time of booking (92.3%), who are in employment (90.4%), of white ethnicity (82.4%) and with normal (<140 mm Hg) systolic blood pressure (98.7%). Of these 65,825 births, 6,343 (9.6%, 99% CI 9.4%-9.9%) were born SGA (Table 1).

Time trends in SES factors are displayed in Supplementary Figure 1. Briefly, less than college (A levels) educational qualification, maternal unemployment and lone motherhood became less prevalent over time (39%, 34% and 9% in 2004 to 22%, 29% and 6% in 2016, respectively), whilst partner unemployment remained relatively stable.

The proportion of SGA births was higher than the average for births to mothers in all disadvantaged SES groups. This includes births to mothers with no university degree (college qualification: 9.0% born SGA, 99% CI 8.5-9.5, secondary school qualification: 11.9% born SGA, 99% CI 11.3-12.5), births to unemployed mothers (11.6% born SGA, 99% CI 11.1-12.2), births to mothers with unemployed partners (14.2% born SGA, 99% CI 13.0-15.4), and births to single mothers (12.4% born SGA, 99% CI 11.3-13.7). Other maternal factors associated with a higher than average rate of SGA include maternal BMI <18.5 kg/m^2 (19.9% born SGA, 99% CI 17.6-22.3), maternal smoking at booking (16.8% born SGA, 99% CI 15.9-17.8) and Asian ethnicity (18.3% born SGA, 99% CI 16.8-19.8).

Table 1 – Maternal/pregnancy characteristics by Small for Gestational Age status (birthweight <10th percentile for gestational age) in the University Hospital Southampton (UHS) maternity population-based cohort (singleton live births 2004-2016, n=65,825)

	SGA		Non-SGA		% SGA	
Characteristics	n	(%)	n	(%)	% SGA	(99% CI)
Highest qualification						
University degree or higher	1,545	(24.4)	17,498	(29.5)	8.1	(7.6 - 8.6)
College	2,371	(37.4)	23,986	(40.4)	9.0	(8.5 - 9.5)
Secondary school or lower	2,424	(38.2)	17,938	(30.2)	11.9	(11.3 - 12.5)
Maternal employment						
Employed	3,868	(61.3)	40,511	(68.6)	8.7	(8.4 - 9.1)
Unemployed	2,438	(38.7)	18,519	(31.4)	11.6	(11.1 - 12.2)
Partner's employment						
Employed	4,969	(85.6)	50,621	(90.9)	8.9	(8.6 - 9.3)
Unemployed	838	(14.4)	5,075	(9.1)	14.2	(13.0 - 15.4)
Partnership						
Partnered	5,706	(90.0)	54,994	(92.5)	9.4	(9.1 - 9.7)
Lone mother	637	(10.0)	4,488	(7.6)	12.4	(11.3 - 13.7)
Maternal BMI						
<18.5 (underweight)	393	(6.2)	1,586	(2.7)	19.9	(17.6 - 22.3)

For peer review only

18.5-24.9 (normal weight)	3,628	(57.2)	30,722	(51.7)	10.6	(10.1 - 11)
25-29.9 (overweight)	1,425	(22.5)	16,070	(27.0)	8.1	(7.6 - 8.7)
30+ (obese)	897	(14.1)	11,104	(18.7)	7.5	(6.9 - 8.1)
Smoking						
Never smoked	3,050	(48.1)	30,760	(51.8)	9.0	(8.6 - 9.4)
Ex-smoker	1,488	(23.5)	19,735	(33.2)	7.0	(6.6 - 7.5)
<10 per day	1,038	(16.4)	5,547	(9.3)	15.8	(14.6-17.0)
10-20 per day	692	(10.9)	3,103	(5.2)	18.2	(16.6 - 19.9)
> 20 per day	71	(1.1)	252	(0.4)	22.0	(16.3 - 28.5)
Maternal age						
18-24	2,000	(31.5)	14,343	(24.1)	12.2	(11.6 - 12.9)
25-34	3,420	(53.9)	35,641	(59.9)	8.8	(8.4 - 9.1)
35-39	754	(11.9)	7,998	(13.5)	8.6	(7.9 - 9.4)
40+	169	(2.7)	1,500	(2.5)	10.1	(8.3 - 12.2)
Previous live births						
None	3,515	(55.4)	25,097	(42.2)	12.3	(11.8 - 12.8)
One or more	2,828	(44.6)	34,385	(57.8)	7.6	(7.2 - 8)
Maternal ethnicity						
White	4,793	(75.6)	49,477	(83.2)	8.8	(8.5 - 9.2)
Mixed	87	(1.4)	720	(1.2)	10.8	(8.1 - 13.9)
Asian	809	(12.8)	3,621	(6.1)	18.3	(16.8 - 19.8)
Black/African/Caribbean	148	(2.3)	1,096	(1.8)	11.9	(9.6 - 14.4)
Chinese	31	(0.5)	427	(0.7)	6.8	(4.1 - 10.4)
Other	116	(1.8)	831	(1.4)	12.2	(9.6 - 15.2)
Not known	359	(5.7)	3,310	(5.6)	9.8	(8.6 - 11.1)
Gestational diabetes						
Not present in current pregnancy	1,475	(97.7)	58,007	(97.5)	9.7	(9.4 - 10.0)
Present in current pregnancy	146	(2.3)	1,475	(2.5)	9.0	(7.3 - 11)
Systolic blood pressure						
<140 mm Hg	6,275	(99.0)	58,578	(98.6)	9.7	(9.4 - 10)
>=140 mm Hg	64	(1.0)	812	(1.4)	7.3	(5.2 - 9.9)
**Overall**	6,343	(100)	59,482	(100)	9.6	(9.4 - 9.9)

	SGA		Non-SGA		
	Mean	(SD)	Mean	(SD)	p-value for t-test
Maternal BMI	24.5	5.2	25.7	5.5	<0.001
Maternal Age	27.9	5.8	28.8	5.5	<0.001
Maternal Systolic blood pressure	107.5	16.2	108.4	17.0	<0.001

For peer review only

For peer review only - http://bmjopen.bmj.com/site/about/guidelines.xhtml

Source: UHS antenatal records for live singleton births (2004-2016). Records with a late antenatal booking (over 24 weeks gestation) were excluded. Variables with missing information include maternal education (68), maternal employment (492) and partner's employment (4,328). The percentage SGA column indicates the percentage of babies born SGA for this characteristic, and the accompanying 99% confidence interval. The t-test indicates whether the mean of each variable differs between those born SGA and non-SGA.

SES differences in SGA risk in the whole cohort

Estimates of the association between maternal SES indicators and risk of SGA are presented in Table 2. The univariable associations between each SES indicator and the risk of SGA are presented in the unadjusted risk row, with all SES indicators being associated with SGA. The size of these effects increase in the first adjusted model (controlling for maternal age, ethnicity, parity, gestational diabetes, gestational hypertension and systolic blood pressure), and attenuate once other SES indicators are controlled for (model 2). Accounting for maternal BMI class had limited impact on effect sizes (model 3). After including maternal smoking all SES inequalities reduced in size (model 4), with the ORs for college qualification compared to university degree (OR 1.10, 99% CI 1.00-1.22) and lone motherhood compared to partnered status attenuating at the 99% level (OR 1.06, 99% CI 0.93-1.20). The full results for model 4 are presented in Supplementary Table 1.

For peer review only

For peer review only - http://bmjopen.bmj.com/site/about/guidelines.xhtml

Table 2– Risk of being born Small for Gestational Age (birthweight <10th percentile for gestational age) by maternal socioeconomic indicator in the University Hospital Southampton (UHS) maternity population-based cohort (singleton live births 2004-2016).

	Mothers with a college qualification vs university degree			Mothers with a school qualification vs university degree			Mothers unemployed at the first antenatal appointment			Lone mothers at the first antenatal appointment		
	OR	99% CI	p	OR	99% CI	p	OR	99% CI	p	OR	99% CI	p
Unadjusted risk	1.12	1.02 - 1.23	0.002	1.53	1.39 - 1.68	<0.001	1.38	1.28 - 1.49	<0.001	1.37	1.22 - 1.54	<0.001
Adjusted risk - Model 1	1.19	1.08 - 1.31	<0.001	1.62	1.46 - 1.78	<0.001	1.55	1.42 - 1.68	<0.001	1.41	1.25 - 1.59	<0.001
Adjusted risk - Model 2	1.16	1.05 - 1.27	<0.001	1.49	1.34 - 1.64	<0.001	1.43	1.31 - 1.55	<0.001	1.26	1.11 - 1.42	<0.001
Adjusted risk - Model 3	1.19	1.08 - 1.32	<0.001	1.53	1.38 - 1.69	<0.001	1.41	1.30 - 1.54	<0.001	1.25	1.10 - 1.41	<0.001
Adjusted risk - Model 4	1.10	1.00 - 1.22	0.011	1.30	1.17 - 1.44	<0.001	1.26	1.16 - 1.38	<0.001	1.06	0.93 - 1.20	0.256

Model 1 adjusts for maternal age, ethnicity, parity, gestational diabetes and systolic blood pressure.

Model 2 is model 1 plus the other SES indicators (n births = 65,331, n mothers = 44,158).

Model 3 is model 2 plus maternal body mass index as a potential mediator (n births = 65,331, n mothers = 44,158).

Model 4 is model 3 plus maternal smoking history as an additional mediator (n births = 65,331, n mothers = 44,158).

OR = odds ratio; CI = confidence interval. In all models the standard errors are adjusted for multiple births per mother.

For peer review only

For peer review only - http://bmjopen.bmj.com/site/about/guidelines.xhtml

In unadjusted estimates presented in Table 3, those born to mothers with unemployed partners at the antenatal booking appointment are 68% more likely to be born SGA (OR 1.68 99% CI 1.51-1.88) in comparison to those born to mothers with employed partners. This association slightly attenuates once maternal education and employment are controlled for (model 2). The association attenuates further once maternal BMI is controlled for (model 3) and remains similar once smoking is accounted for (model 4 OR 1.27, 99% CI 1.13-1.43). The full results for model 4 are presented in Supplementary Table 2.

For peer review only

For peer review only - http://bmjopen.bmj.com/site/about/guidelines.xhtml

Table 3 – Risk of being born Small for Gestational Age (birthweight <10th percentile for gestational age) by partner's employment status in the University Hospital Southampton (UHS) maternity population-based cohort (singleton live births 2004-2016).

	Mothers with an unemployed partner		
	OR	99% CI	p
Unadjusted risk	1.68	1.51 - 1.88	<0.001
Adjusted risk - Model 1	1.69	1.51 - 1.89	<0.001
Adjusted risk - Model 2	1.48	1.32 - 1.66	<0.001
Adjusted risk - Model 3	1.49	1.33 - 1.68	<0.001
Adjusted risk - Model 4	1.27	1.13 - 1.43	<0.001

Model 1 adjusts for maternal age, ethnicity, parity, gestational diabetes and systolic blood pressure.

Model 2 is model 1 plus the other two SES indicators (n births = 61,170, n mothers = 42,217).

Model 3 is model 2 plus maternal body mass index as a potential mediator (n births = 61,170, n mothers = 42,217).

Model 4 is model 3 plus maternal smoking history as an additional mediator (n births = 61,170, n mothers = 42,217).

OR = odds ratio; CI = confidence interval. In all models the standard errors are adjusted for multiple births per mother.

For peer review only

As a sensitivity analysis, we repeated the modelling for a subgroup of women who were resident in Southampton at the time of delivery to address the potential that the whole sample results may be biased by including potential high-risk referrals from other regions to this specialised maternity centre. The geographical residence data (lower super output areas) were retrieved from health visitor records, and linked to births in this cohort as part of a bigger research project utilising an anonymised linked mother-child dataset. Each child in England and Wales is followed up by health visitor teams for at least 5 key appointments which start at 28 weeks into pregnancy [40], so this sub-sample is unlikely to be affected by selection bias. From the sample of 65,412 births, 32,147 (49%) were resident in Southampton at this 28 week appointment. In a model that adjusts for all confounders, maternal BMI category and smoking, the confidence intervals for the SES factors overlap in the Southampton only sample, and those results presented in Tables 2 and 3 (see Supplementary Table 3 for full results), indicating largely similar risk estimates between the two samples.

Time trend in SES inequalities in the risk of SGA between 2004 and 2016

To test whether SES inequalities are narrowing or widening over time, interactions between year (continuous) and SES ('slope') were included to model 4 in Table 2 and Table 3, and expressed as ORs. A positive slope OR indicates that the disadvantaged SES group are becoming at greater risk of SGA relative to the advantaged group over calendar year, and vice versa for a negative effect.

For peer review only

List of Figures:

Figure 1a-d displays the adjusted ORs for each SES indicator by year in the cohort (UHS), and the accompanying p-value for the slope over calendar year. The slopes for maternal college and school qualifications (OR 1.00, 99% CI 0.98-1.03; OR 1.00, 99% CI 0.97-1.02), maternal employment (OR 1.00, 99% CI 0.98-1.02), lone motherhood (OR 1.00, 99% CI 0.98-1.02) and partner unemployment (OR 1.00, 99% CI 0.97-1.03) were not statistically significant. Models using a binary indicator for pre- and post-2008 (2003-2008 and 2009-2016) showed no significant differences in the magnitude of SES inequalities (results not shown).

For peer review only

For peer review only - http://bmjopen.bmj.com/site/about/guidelines.xhtml

<Figure 1>

For peer review only

For peer review only - http://bmjopen.bmj.com/site/about/guidelines.xhtml

SES differences in SGA risk by maternal parity status

For this analysis, the sample was restricted to the first antenatal care record per mother included in our dataset with no missing information (21,200 records dropped, with a new total of 44,212). Interaction terms between each SES indicator and parity (accounting for control variables) were conducted utilising this sample showing a significant interaction between maternal employment status and SGA (p=0.010). We then stratified the sample by parity (n primiparous (0 previous live births) = 28,519; n multiparous (1 or more previous live births) = 15,693). The modelling strategy used in the first analysis is repeated on these sub-samples to assess the risk estimates by parity.

The association between secondary school qualification versus university degree and the risk of SGA appeared less pronounced among primiparous (OR 1.26, 99% CI 1.11-1.44) than multiparous women (OR 1.38, CI 1.09-1.75). Maternal unemployment (relative to mothers who were employed) was associated with higher risk of SGA in primiparous women (aOR 1.29, 99% CI 1.14-1.47) than among multiparous women (aOR 1.18, 99% CI 1.00-1.39). The associations between college qualification versus university degree, and lone motherhood versus partnered status, with SGA risk appeared to be mediated by smoking in all sub-samples (Table 4).

Table 5Table 5 displays the results for partner's employment (total n mothers = 42,265; 26,838 primiparous, 15,427 multiparous). The association between partner's employment and risk of SGA appeared to be mediated by maternal smoking among multiparous women (aOR 1.16, CI 0.93-1.40), but not primiparous women (aOR 1.34, CI 1.13-1.58). The estimates of SES differences in the risk of SGA were similar in the reduced sample (Tables 4 and 5) and the whole sample (Tables 2 and 3).

To summarise the above models, both maternal and partner's employment status appeared to be more strongly associated with SGA risk in primiparous than multiparous women, and the reverse is true for maternal educational attainment.

For peer review only

For peer review only - http://bmjopen.bmj.com/site/about/guidelines.xhtml

Table 4 - Risk of being born Small for Gestational Age (birthweight <10th percentile for gestational age) by maternal socioeconomic indicator and stratified by parity in the University Hospital Southampton (UHS) maternity population-based cohort (singleton live births 2004-2016, one live birth per mother)

		Mothers with a college qualification vs university degree			Mothers with a school qualification vs university degree			Mothers unemployed at the first antenatal appointment			Lone mothers at the first antenatal appointment		
Sample	Model	OR	99% CI	p	OR	99% CI	p	OR	99% CI	p	OR	99% CI	p
Whole sample n mothers = 44,158	Unadjusted risk	1.08	0.98 - 1.20	0.036	1.41	1.28 - 1.56	<0.001	1.37	1.26 - 1.49	<0.001	1.33	1.17 - 1.52	<0.001
	Adjusted risk - Model 1	1.17	1.05 - 1.30	<0.001	1.54	1.38 - 1.71	<0.001	1.48	1.35 - 1.63	<0.001	1.40	1.22 - 1.60	<0.001
	Adjusted risk - Model 2	1.15	1.04 - 1.28	<0.001	1.45	1.30 - 1.62	<0.001	1.39	1.26 - 1.53	<0.001	1.27	1.10 - 1.45	<0.001
	Adjusted risk - Model 3	1.18	1.06 - 1.31	<0.001	1.48	1.33 - 1.66	<0.001	1.37	1.24 - 1.51	<0.001	1.21	1.02 - 1.44	0.004
	Adjusted risk - Model 4	1.10	0.99 - 1.23	0.020	1.28	1.15 - 1.44	<0.001	1.24	1.12 - 1.37	<0.001	1.09	0.95 - 1.25	0.115
Primiparous women only n births = 28,469	Unadjusted risk	1.11	0.99 - 1.24	0.020	1.48	1.31 - 1.67	<0.001	1.78	1.59 - 1.99	<0.001	1.78	1.59 - 1.99	<0.001
	Adjusted risk - Model 1	1.13	1.00 - 1.27	0.009	1.43	1.26 - 1.63	<0.001	1.50	1.33 - 1.69	<0.001	1.33	1.12 - 1.57	<0.001
	Adjusted risk - Model 2	1.12	0.99 - 1.26	0.014	1.37	1.20 - 1.56	<0.001	1.42	1.25 - 1.61	<0.001	1.21	1.02 - 1.44	0.004
	Adjusted risk - Model 3	1.14	1.01 - 1.29	0.004	1.39	1.22 - 1.58	<0.001	1.39	1.23 - 1.58	<0.001	1.37	1.09 - 1.72	<0.001
	Adjusted risk - Model 4	1.09	0.96 - 1.23	0.081	1.26	1.10 - 1.44	<0.001	1.29	1.13 - 1.46	<0.001	1.08	0.91 - 1.29	0.252
Multiparous women only n births = 15,699	Unadjusted risk	1.24	0.99 - 1.55	0.014	1.80	1.46 - 2.23	<0.001	1.52	1.31 - 1.77	<0.001	1.52	1.31 - 1.77	<0.001
	Adjusted risk - Model 1	1.33	1.06 - 1.68	0.001	1.87	1.50 - 2.33	<0.001	1.44	1.23 - 1.69	<0.001	1.55	1.23 - 1.94	<0.001
	Adjusted risk - Model 2	1.29	1.03 - 1.63	0.004	1.72	1.37 - 2.15	<0.001	1.31	1.11 - 1.53	<0.001	1.27	1.10 - 1.45	<0.001
	Adjusted risk - Model 3	1.35	1.07 - 1.70	0.001	1.81	1.44 - 2.27	<0.001	1.31	1.11 - 1.54	<0.001	1.37	1.09 - 1.73	<0.001
	Adjusted risk - Model 4	1.18	0.93 - 1.50	0.065	1.38	1.09 - 1.75	<0.001	1.17	0.99 - 1.38	0.015	1.10	0.87 - 1.39	0.300

Model 1 adjusts for maternal age, ethnicity, gestational diabetes and systolic blood pressure.

Model 2 is model 1 plus the other two SES indicators (e.g. the maternal unemployment column is adjusted for maternal education and partnership).

Model 3 is model 2 plus maternal body mass index as a potential mediator.

Model 4 is model 3 plus maternal smoking history as an additional mediator.

OR = odds ratio; CI = confidence interval. All models for the whole sample are adjusted for parity.

For peer review only - http://bmjopen.bmj.com/site/about/guidelines.xhtml

Table 5 - Risk of being born Small for Gestational Age (birthweight <10th percentile for gestational age) by partner's employment and stratified by parity in the University Hospital Southampton maternity population-based cohort (singleton live births 2004-2016)

		Unemployed partner at first antenatal appointment		
Sample	Model	OR	99% CI	p
Whole sample<br>n mothers = 42,217	Unadjusted risk	1.61	1.42 - 1.81	<0.001
	Adjusted risk - Model 1	1.61	1.42 - 1.83	<0.001
	Adjusted risk - Model 2	1.44	1.26 - 1.64	<0.001
	Adjusted risk - Model 3	1.44	1.27 - 1.65	<0.001
	Adjusted risk - Model 4	1.27	1.11 - 1.45	<0.001
Primiparous women only<br>n births = 26,792	Unadjusted risk	1.75	1.50 - 2.05	<0.001
	Adjusted risk - Model 1	1.60	1.36 - 1.88	<0.001
	Adjusted risk - Model 2	1.43	1.21 - 1.70	<0.001
	Adjusted risk - Model 3	1.44	1.22 - 1.71	<0.001
	Adjusted risk - Model 4	1.33	1.12 - 1.58	<0.001
Multiparous women only<br>n births = 15,425	Unadjusted risk	1.67	1.37 - 2.03	<0.001
	Adjusted risk - Model 1	1.63	1.33 - 1.99	<0.001
	Adjusted risk - Model 2	1.43	1.16 - 1.76	<0.001
	Adjusted risk - Model 3	1.45	1.17 - 1.78	<0.001
	Adjusted risk - Model 4	1.16	0.94 - 1.44	0.070

Model 1 adjusts for maternal age, ethnicity, gestational diabetes and systolic blood pressure.

Model 2 is model 1 plus maternal education and employment

Model 3 is model 2 plus maternal body mass index as a potential mediator.

Model 4 is model 3 plus maternal smoking history as an additional mediator

OR = odds ratio; CI = confidence interval. All models for the whole sample are adjusted for parity.

For peer review only

Discussion

In this analysis of routine maternity healthcare data from a regional hospital in Southampton, UK, multivariable logistic regression was used to examine the relationship between SES indicators (education, employment and partnership) and SGA, and whether these relationships are stable over time and different by parity. Educational attainment and employment (of the mother and her partner) were independently associated with the risk of SGA, although differences between the association between single motherhood and SGA were attenuated by adjusting for smoking status. SES differences in the risk of SGA were stable over the study period (2004-2016). The strength of these SES differences varied between mothers at their first and higher order births. Maternal and partner unemployment were associated with a higher risk of SGA in mothers with no previous live births, with lower educational qualification being more strongly associated with SGA risk in mothers with previous live births.

Comparison with other studies

The evidence for SES inequalities by maternal educational attainment, employment and partner's employment in the risk of SGA is consistent with the literature, and the third analysis shows that these associations remain robust after limiting the sample to one record per mother. Within a systematic review of socioeconomic disparities in birth outcomes conducted in 2010 [7], 6 of the 9 (66%) studies of SGA and maternal education reported a significant association, in addition to single studies finding an association for maternal [41] and paternal employment [42]. Part of the complexity in the relationship between maternal SES and SGA results from many analyses using only one measure of SES, with maternal education [15,43] and employment [44] being the main indicators used. Factors related to the mother's partner are usually excluded, due to a lack of appropriate data or small sample sizes, despite the potential of these factors to influence pregnancy conditions and outcomes [45]. Whether the mother has a partner or not is largely overlooked as a risk factor in this area, with the exception of Kleijer et al [46], who found that single mothers are at higher risk of SGA. The final estimates of SES inequalities in this study are adjusted for other SES indicators, suggesting that there are multiple pathways through which SES is linked to gestational growth.

Since the publication of Blumenshine *et al*'s systematic review [6] there has been an increased focus on how SES differences in weight outcomes at birth and during early life may be mediated through maternal BMI and smoking. In a Dutch cohort, maternal smoking and height during pregnancy were reported to explain 75% of the difference in risk of SGA between mothers with low and high education [15]. In an Australian cohort maternal smoking and the BMI of both parents were reported to explain 83.5% of SES differences in their children's BMI Z-score at age 10-11 years [47]. In the present analysis, accounting for maternal smoking reduced the magnitude of the SGA risk difference by SES from a 36% increase in risk to 20% among mothers without a university degree, and from a 48% to 31% increase in risk among unemployed mothers. Maternal smoking also explained the relatively high risk of SGA among single mothers. This attenuation corroborates previous research indicating that single mothers are more likely to smoke, and that this may be related to the level of stress that they report, relative to partnered mothers [48]. Single mothers may be relying on smoking as a means of stress relief or management during pregnancy, and smoking cessation and support programmes may be effective in reducing inequalities in birth outcomes as a result.

To our knowledge, there has been no analysis of socioeconomic inequality time trends in SGA from the mid-2000s onwards in England. Inequalities in birthweight (adjusting for gestational age) were

For peer review only - http://bmjopen.bmj.com/site/about/guidelines.xhtml

stable between 1961 and 2000 in a regional city-based study in North East England [30], and the same is found between 2004 and 2016 in this study. The stability of SES inequalities in SGA implies that further interventions and initiatives are required to narrow SES inequalities in SGA births.

Our hypothesis was that the extent of SES inequalities in the risk of SGA may differ by parity, as the birth of the first child is a period which brings about significant physiological, lifestyle and social changes, in addition to postpartum weight retention [18]. An analysis of birth register data in Norway found that mothers who had several SGA births were characterized by low educational attainment and partners employed in low SES occupations [19]. In the present analysis, the strength of the association between SES indicators and the risk of SGA varied between primiparous and multiparous women, with education inequalities being greater for multiparous women, and employment inequalities being greater for primiparous women. The explanation may be that more advantaged women are economically able to leave the workforce after their first birth when planning further pregnancies (thus attenuating the differences between those in and outside employment when having subsequent births), whilst educational differences in terms of health behaviours, health literacy and mental wellbeing are risk factors of having repeat or new SGA outcomes [49]. This group may benefit from additional support following the birth of their first baby to promote mental and physical wellbeing, access appropriate services, enhance health literacy and facilitate healthy behaviours.

Strengths and limitations of the study

This study benefits from a large regionally-representative sample over many years. The exposure measures are prospectively collected in the course of routine antenatal care. As data from the local hospital system are used, there is no selection bias which may arise from participation in a research cohort, and the sample is therefore representative of all those receiving care under this NHS site. The outcome (SGA) is derived from birthweight, which is objectively measured by a health professional at birth. The most recent birth centiles for England and Wales were used [29] to reflect changes in birth weight since the oft-used 1990 birth centiles [50]. The measures of SES used are also collected within the usual course of NHS care before birth, so the results may be used to inform risk stratification interventions at or following the booking appointment to curtail SGA births and other associated adverse health outcomes. The antenatal booking appointment is a critical point for intervention as health professionals see all mothers receiving care under the NHS. The results herein find that women who report low educational qualifications, are unemployed, or their partner is unemployed at this stage are at higher risk of SGA delivery. These groups, as well as women with no partners and/or other social support at the time of the booking appointment, may then be referred for additional support to minimize the risk of an SGA birth and other adverse maternal and health outcomes. A limitation of our dataset is that such processes were not electronically recorded and hence not includes in our analyses. In addition, as this research is based on a cohort, we cannot infer that SES has a causal effect on SGA risk.

Some potential risk factors were not adjusted for in this study due to inconsistency of data for those specific variables as captured routinely in antenatal care, including diet during pregnancy and alcohol intake. These factors may also mediate the effect of SES on SGA risk, wherein disadvantaged SES groups could be more likely to engage in risky health behaviours. Other important SES factors such as sector of employment and income have been related to SGA outcomes in previous research [9], but are also not routinely collected in antenatal practice. The same is true for other measures of deprivation level such as housing, transportation methods and access to healthcare and other facilities.

For peer review only - http://bmjopen.bmj.com/site/about/guidelines.xhtml

For the parity analysis, we did not account for the length of the interpregnancy interval which has been related to birth outcomes previously [51,52]. It was not possible to control for this in our study due to a lack of data on this variable in the whole sample, because we have included the first pregnancy in the database per mother and some multiparous mothers would have given birth before the study period, or at other hospitals, hence this information is lacking for them. In addition, this analysis did not account for characteristics of the residential environments mothers lived in during pregnancy. Systematic reviews indicate that social, built and air characteristics of the environment experienced during pregnancy are strongly associated with birth outcomes [6,53], and this will be addressed in a follow-up study on the associations between environmental characteristics and birth outcomes for the cohort.

As the data used in this study are limited to a hospital serving the city of Southampton and the surrounding region, the results may not apply to hospitals serving populations with differing characteristics. Southampton is a provincial urban city which is more deprived than the average Local Authority in England, although the surrounding area (Hampshire) is relatively affluent [54]. Southampton has a similar ethnicity profile to the rest of England and Wales [55], but with a relatively large university student population, and women in Southampton are underrepresented in managerial, administrative and professional occupations, relative to others in England [56]. As a result, findings from this study may not be replicated using healthcare records in areas with predominantly rural populations, or areas with non-student and managerial populations.

The UHS is a regional maternity centre to which high-risk pregnancies may be referred leading to potential over-representation of them. We have addressed this through excluding pregnancies booking in the UHS system after 24 weeks gestation. Mothers attending later than this date may have been referred to UHS due to their pregnancy being identified as high-risk**.** We have also conducted sensitivity analyses restricting the sample to those who were living in the city of Southampton at the time of birth, and there was no significant difference in effect sizes. The proportion of mothers in employment (64%) and with a university degree (28%) were similar in our cohort in comparison to Census figures for Southampton women aged 20-39 (69%) and 16-34 (29%), respectively, indicating that our sample is representative of the catchment area for the UHS [57,58].

Implications for research and practice

The persistence of educational and employment inequalities in the risk of SGA found within this study justifies further interventions and initiatives in order to narrow SES inequalities in the risk of SGA, and subsequently their long-term adverse health impact. The antenatal booking appointment offers an opportune moment for risk stratification and signposting of additional support for women with low educational qualification, in unemployed households and low social support. Smoking appeared as a potential mediator for SES inequalities in this study, despite support in smoking cessation being offered in the course of NHS care [59]. This suggests that further support is required for mothers of low SES, and pre- and interconception programmes may have the added benefit of reducing the extent of SES inequalities in SGA, in addition to overall SGA rates. For research, this study aligns with recent calls to incorporate paternal/partner influences in developmental health research [45], in that similar levels of SGA risk are found for maternal and partner unemployment. Research in this area should adopt a more family-centred approach in relation to offspring health outcomes, taking into account contributing exposures from others within the household structure (partners and siblings).

For peer review only - http://bmjopen.bmj.com/site/about/guidelines.xhtml

Conclusions

This study confirms that socioeconomic status indicators, including educational attainment, employment status and single motherhood, are strongly and independently associated with the risk of small for gestational age birth, and they are not narrowing over time. Maternal smoking appears to play a significant role in these inequalities, particularly for lone mothers. However, the associations between educational attainment and employment status with SGA risk remain strong even after accounting for maternal smoking and BMI. Inequalities in SGA risk by maternal educational attainment appear greater for multiparous compared to primiparous women, while the opposite is true by maternal and partner employment status. Further research is needed to identify critical windows of opportunity (preconception/pregnancy/interconception) and effective interventions in order to narrow these inequalities. Prevention programmes targeting socioeconomically disadvantaged women which incorporate smoking cessation and social support are vital to tackling health inequalities in SGA.

For peer review only

What is already known on this topic

- Babies born to mothers in low socioeconomic status (SES) are at higher risk of being born small for gestational age (SGA).

- These SES inequalities were found to be stable between 1961 and 2000 in a previous English study.

- The relationship between maternal SES and SGA is linked to a higher prevalence of smoking and maternal underweight among mothers in low SES.

What this study adds

- Indicators of parental SES (maternal education, maternal and partner employment) are independently associated with the risk of being born SGA with some variation in the magnitude of risk between primiparous and multiparous women, while the risk difference between lone and partnered mothers is attenuated by accounting for maternal smoking.

-These SES inequalities remained stable between 2004 and 2016 in this English population-based cohort.

For peer review only

Footnotes

Contributorship statement

NAA is the Principal Investigator of the project, and acts as the guarantor of this study. SW, NZ, PR, DS, DC, NM, MH and NAA contributed to study conception and design. NM provided input on the statistical analysis for this study. SW conducted the statistical analyses, and drafted the initial report. NZ checked the accuracy of the reported estimates from the statistical models. All authors contributed to interpretation of data and revising the manuscript critically for important intellectual content. All authors read and approved the final manuscript.

Funding

This research is supported by an Academy of Medical Sciences and Wellcome Trust grant to NAA [Grant no: AMS_HOP001\1060], and the National Institute for Health Research through the NIHR Southampton Biomedical Research Centre. The research funders had no input on research design or manuscript drafting. MAH is supported by the British Heart Foundation and the National Institute for Health Research through the NIHR Southampton Biomedical Research Centre.

Acknowledgments

We thank Ravita Taheem (Senior Public Health Practitioner, Southampton City Council) for her input in the conception of this paper, David Cable (Electronic Patient Records Implementation and Service Manager) and Florina Borca (Senior Information Analyst for R&D, NIHR Southampton Biomedical Research Centre) at University Hospital Southampton NHS Foundation Trust for their support in extracting the data used in this study.

Competing interests

All authors have completed the ICMJE uniform disclosure form at www.icmje.org/coi_disclosure.pdf and declare: NAA had financial support from the Academy of Medical Sciences/Wellcome Trust and the NIHR Southampton Biomedical Research Centre for the submitted work; no financial relationships with any organisations that might have an interest in the submitted work in the previous three years; NAA is a member of the National Institute for Health and Care Excellence (NICE) Antenatal Care Guideline Committee; no other relationships or activities that could appear to have influenced the submitted work.

Ethical approval

This study used anonymised antenatal record data supplied by University Hospital Southampton Trust. This analysis forms part of a research project reviewed and approved by the University of Southampton Faculty of Medicine Ethics Committee (ref 24433) and the National Health Service Health Research Authority (ref 242031).

For peer review only

For peer review only - http://bmjopen.bmj.com/site/about/guidelines.xhtml

Data availability

The authors' ethical approval from the Faculty of Medicine Ethics Committee, University of Southampton (Reference number 24433) restricts public sharing of the data used in this study. The data owner is University Hospital Southampton NHS Trust. Please contact NAA to request data access beyond that included in the manuscript. Further ethical and research governance approval may be required.

Transparency

The first author, SW, affirms that the manuscript is an honest, accurate, and transparent account of the study being reported.

For peer review only

For peer review only - http://bmjopen.bmj.com/site/about/guidelines.xhtml

References

1 Mendez-Figueroa H, Truong V, Pedroza C, *et al.* Morbidity and mortality with small for gestational age: Secondary analysis of nine MFMU network studies. Am. J. Obstet. Gynecol. 2016;**214**:S320–1. doi:10.1055/s-0036-1586502

2 Reilly JJ, Armstrong J, Emmett P, *et al.* Early life risk factors for obesity in childhood. *Bmj* 2005;**331**:454.1. doi:10.1136/bmj.331.7514.454

3 Barker DJP. The Developmental Origins of Adult Disease. *J Am Coll Nutr* 2004;**23**:588S–595S. doi:10.1080/07315724.2004.10719428

4 McCowan L, Horgan RP. Risk factors for small for gestational age infants. *Best Pract Res Clin Obstet Gynaecol* 2009;**23**:779–93. doi:10.1016/j.bpobgyn.2009.06.003

5 Mitchell EA, Robinson E, Clark PM, *et al.* Maternal nutritional risk factors for small for gestational age babies in a developed country: A case-control study. *Arch Dis Child Fetal Neonatal Ed* 2004;**89**:F431–5. doi:10.1136/adc.2003.036970

6 Vos AA, Posthumus AG, Bonsel GJ, *et al.* Deprived neighborhoods and adverse perinatal outcome: a systematic review and meta-analysis. *Acta Obstet Gynecol Scand* 2014;**93**:727–40. doi:10.1111/aogs.12430

7 Blumenshine P, Egerter S, Barclay CJ, *et al.* Socioeconomic Disparities in Adverse Birth Outcomes: A Systematic Review. *Am J Prev Med* 2010;**39**:263–72. doi:10.1016/j.amepre.2010.05.012

8 Ruiz M, Goldblatt P, Morrison J, *et al.* Mother's education and the risk of preterm and small for gestational age birth: a DRIVERS meta-analysis of 12 European cohorts. *J Epidemiol Community Health* 2015;**69**:826 LP-833. doi:10.1136/jech-2014-205387

9 Sadovsky ADI, Matijasevich A, Santos IS, *et al.* LBW and IUGR temporal trend in 4 population-based birth cohorts: The role of economic inequality. *BMC Pediatr* 2016;**16**. doi:10.1186/s12887-016-0656-0

10 Gao W, Paterson J, Carter S, *et al.* Risk factors for preterm and small-for-gestational-age babies: a cohort from the Pacific Islands Families Study. *J Paediatr Child Health* 2006;**42**:785–92. doi:10.1111/j.1440-1754.2006.00978.x

11 Luo Z-C, Wilkins R, Kramer MS, *et al.* Effect of neighbourhood income and maternal education on birth outcomes: a population-based study. *C Can Med Assoc J* 2006;**174**:1415–20. doi:10.1503/cmaj.051096

12 Masi CM, Hawkley LC, Piotrowski ZH, *et al.* Neighborhood economic disadvantage, violent crime, group density, and pregnancy outcomes in a diverse, urban population. *Soc Sci Med* 2007;**65**:2440–57. doi:10.1016/j.socscimed.2007.07.014

13 Pevalin DJ, Wade TJ, Brannigan A, *et al.* Beyond biology: The social context of prenatal behaviour and birth outcomes. *Soz Praventivmed* 2001;**46**:233–9. doi:10.1007/BF01593178

14 Savitz DA, Kaufman JS, Dole N, *et al.* Poverty, education, race, and pregnancy outcome. *Ethn Dis* 2004;**14**:322–9.

15 van den Berg G, van Eijsden M, Galindo-Garre F, *et al.* Smoking overrules many other risk factors for small for gestational age birth in less educated mothers. *Early Hum Dev* 2013;**89**:497–501. doi:10.1016/j.earlhumdev.2013.03.007

16 Love C, David RJ, Rankin KM, *et al.* Exploring Weathering: Effects of Lifelong Economic

For peer review only - http://bmjopen.bmj.com/site/about/guidelines.xhtml

Environment and Maternal Age on Low Birth Weight, Small for Gestational Age, and Preterm Birth in African-American and White Women. *Am J Epidemiol* 2010;**172**:127–34. doi:10.1093/aje/kwq109

17 Taylor-Robinson D, Agarwal U, Diggle PJ, *et al.* Quantifying the Impact of Deprivation on Preterm Births: A Retrospective Cohort Study. *PLoS One* 2011;**6**:e23163. doi:10.1371/journal.pone.0023163

18 Hollis JL, Crozier SR, Inskip HM, *et al.* Modifiable risk factors of maternal postpartum weight retention: An analysis of their combined impact and potential opportunities for prevention. *Int J Obes* 2017;**41**:1091–8. doi:10.1038/ijo.2017.78

19 Bakketeig LS, Bjerkedal T, Hoffman HJ. Small-for-gestational age births in successive pregnancy outcomes: results from a longitudinal study of births in Norway. *Early Hum Dev* 1986;**14**:187–200. doi:10.1016/0378-3782(86)90180-5

20 Milgrom J, Gemmill AW, Bilszta JL, *et al.* Antenatal risk factors for postnatal depression: A large prospective study. *J Affect Disord* 2008;**108**:147–57. doi:10.1016/j.jad.2007.10.014

21 Patel V, Rodrigues M, DeSouza N. Gender, poverty, and postnatal depression: A study of mothers in Goa, India. *Am J Psychiatry* 2002;**159**:43–7. doi:10.1176/appi.ajp.159.1.43

22 Local Government Association. Fit for and during pregnancy: a key role for local government. 2018.https://www.local.gov.uk/sites/default/files/documents/15.52 Fit for and during pregnancy_03.pdf (accessed 4 Jun 2019).

23 Public Health England. Public Health Outcomes Framework for England. 2019.https://web.archive.org/web/20190406232547/https://fingertips.phe.org.uk/profile/public-health-outcomes-framework (accessed 4 Jun 2019).

24 Kana MA, Correia S, Peleteiro B, *et al.* Impact of the global financial crisis on low birth weight in Portugal: a time-trend analysis. *BMJ Glob Heal* 2017;**2**:e000147. doi:10.1136/bmjgh-2016-000147

25 Richard B, Monica CD, Costas M, *et al.* Female Labor Supply, Human Capital, and Welfare Reform. *Econometrica* 2016;**84**:1705–53. doi:10.3982/ECTA11576

26 Rafferty A, Wiggan J. The Time-related Underemployment of Lone Parents during Welfare Reform, Recession and Austerity: A Challenge to In-work Conditionality? *Soc Policy Adm* 2017;**51**:511–38. doi:10.1111/spol.12190

27 Jofre-Bonet M, Serra-Sastre V, Vandoros S. The impact of the Great Recession on health-related risk factors, behaviour and outcomes in England. *Soc Sci Med* 2018;**197**:213–25. doi:10.1016/j.socscimed.2017.12.010

28 National Institute for Clinical Excellence. Antenatal care for uncomplicated pregnancies: schedule of appointments. 2012.https://pathways.nice.org.uk/pathways/antenatal-care-for-uncomplicated-pregnancies/antenatal-care-for-uncomplicated-pregnancies-schedule-of-appointments.pdf (accessed 4 Jun 2019).

29 Norris T, Seaton SE, Manktelow BN, *et al.* Updated birth weight centiles for England and Wales. *Arch Dis Child Fetal Neonatal Ed* 2018;**103**:F577–82. doi:10.1136/archdischild-2017-313452

30 Glinianaia S V, Ghosh R, Rankin J, *et al.* No improvement in socioeconomic inequalities in birthweight and preterm birth over four decades: a population-based cohort study. *BMC Public Health* 2013;**13**:345. doi:10.1186/1471-2458-13-345

For peer review only

For peer review only - http://bmjopen.bmj.com/site/about/guidelines.xhtml

31 World Health Organization. Physical status: The use and interpretation of anthropometry: Report of a WHO Expert Committee. World Heal. Organ. - Tech. Rep. Ser. 1995.

32 Royal College of Obstetricians and Gynaecologists. The investigation and management of the Small-for-Gestational-Age Fetus. Green-top Guidel. No. 31. 2014.https://www.rcog.org.uk/globalassets/documents/guidelines/gtg_31.pdf (accessed 4 Jun 2019).

33 National Maternity and Perinatal Audit Team. *National Maternity and Perinatal Audit: Clinical report 2017*. London: : Royal College of Obstetricians and Gynaecologists 2017.

34 Kozuki N, Lee ACC, Silveira MF, *et al.* The associations of parity and maternal age with small-for-gestational-age, preterm, and neonatal and infant mortality: a meta-analysis. *BMC Public Health* 2013;**13**:S2–S2. doi:10.1186/1471-2458-13-S3-S2

35 Block-Abraham DM, Adamovich D, Turan OM, *et al.* Maternal blood pressures during pregnancy and the risk of delivering a small-for-gestational-age neonate. *Hypertens pregnancy* 2016;**35**:350–60. doi:10.3109/10641955.2016.1150487

36 World Health Organization. Body mass index. 2019.http://www.euro.who.int/en/health-topics/disease-prevention/nutrition/a-healthy-lifestyle/body-mass-index-bmi (accessed 4 Jun 2019).

37 Ranganathan P, Pramesh CS, Buyse M. Common pitfalls in statistical analysis: The perils of multiple testing. *Perspect Clin Res* 2016;**7**:106–7. doi:10.4103/2229-3485.179436

38 Baron RM, Kenny DA. The moderator-mediator variable distinction in social psychological research: conceptual, strategic, and statistical considerations. *J Pers Soc Psychol* 1986;**51**:1173–82.

39 Galobardes B, Costanza MC, Bernstein MS, *et al.* Trends in Risk Factors for Lifestyle-Related Diseases by Socioeconomic Position in Geneva, Switzerland, 1993–2000: Health Inequalities Persist. *Am J Public Health* 2003;**93**:1302–9. doi:10.2105/AJPH.93.8.1302

40 Department of Health. Healthy Child Programme: Pregnancy and the first five years of life. 2009.

41 Gissler M, Rahkonen O, Arntzen A, *et al.* Trends in socioeconomic differences in Finnish perinatal health 1991-2006. *J Epidemiol Community Health* 2009;**63**:420–5. doi:10.1136/jech.2008.079921

42 L. F, A.H. L. Social class inequalities in perinatal outcomes: Scotland 1980-2000. *J Epidemiol Community Health* 2006;**60**:31–6. doi:10.1136/jech.2005.038380

43 Agyemang C, Vrijkotte TGM, Droomers M, *et al.* The effect of neighbourhood income and deprivation on pregnancy outcomes in Amsterdam, The Netherlands. *J Epidemiol Community Health* 2009;**63**:755 LP-760. doi:10.1136/jech.2008.080408

44 Räisänen S, Gissler M, Sankilampi U, *et al.* Contribution of socioeconomic status to the risk of small for gestational age infants – a population-based study of 1,390,165 singleton live births in Finland. *Int J Equity Health* 2013;**12**:28. doi:10.1186/1475-9276-12-28

45 Sharp GC, Lawlor DA, Richardson SS. It's the mother!: How assumptions about the causal primacy of maternal effects influence research on the developmental origins of health and disease. *Soc Sci Med* 2018;**213**:20–7. doi:10.1016/j.socscimed.2018.07.035

46 Kleijer ME, Dekker GA, Heard AR. Risk factors for intrauterine growth restriction in a socio-economically disadvantaged region. *J Matern Neonatal Med* 2005;**18**:23–30.

For peer review only

For peer review only - http://bmjopen.bmj.com/site/about/guidelines.xhtml

doi:10.1080/14767050500127674

47 Iguacel I, Chung A, Gearon E, *et al.* Influence of early-life risk factors on socioeconomic inequalities in weight gain. *J Public Health (Oxf)* 2018;**40**:e447–55. doi:10.1093/pubmed/fdy056

48 Sperlich S, Maina MN, Noeres D. The effect of psychosocial stress on single mothers' smoking. *BMC Public Health* 2013;**13**:1125. doi:10.1186/1471-2458-13-1125

49 Güneş PM. The role of maternal education in child health: Evidence from a compulsory schooling law. *Econ Educ Rev* 2015;**47**:1–16. doi:10.1016/j.econedurev.2015.02.008

50 Cole TJ, Freeman J V., Preece MA. British 1990 growth reference centiles for weight, height, body mass index and head circumference fitted by maximum penalized likelihood. *Stat Med* 1998;**17**:407–29. doi:10.1002/(SICI)1097-0258(19980228)17:4<407::AID-SIM742>3.0.CO;2-L

51 Kozuki N, Lee AC, Silveira MF, *et al.* The associations of birth intervals with small-for-gestational-age, preterm, and neonatal and infant mortality: A meta-analysis. BMC Public Health. 2013. doi:10.1186/1471-2458-13-S3-S3

52 Ziauddeen N, Roderick PJ, Macklon N, *et al.* Is the duration of the preceding inter-pregnancy interval associated with offspring's size at birth?–analysis of a UK population-based cohort. *J Epidemiol Community Heal* 2018;**1**:72. doi:10.1136/jech-2018-SSMabstracts.89

53 Li X, Huang S, Jiao A, *et al.* Association between ambient fine particulate matter and preterm birth or term low birth weight: An updated systematic review and meta-analysis. *Environ Pollut* 2017;**227**:596–605. doi:10.1016/j.envpol.2017.03.055

54 Department for Communities and Local Government. English Indices of Deprivation 2015. 2015.https://www.gov.uk/government/uploads/system/uploads/attachment_data/file/467764/File_1_ID_2015_Index_of_Multiple_Deprivation.xlsx (accessed 4 Jun 2019).

55 Office for National Statistics. Ethnicity and National Identity in England and Wales 2011. 2012.https://www.ons.gov.uk/peoplepopulationandcommunity/culturalidentity/ethnicity/articles/ethnicityandnationalidentityinenglandandwales/2012-12-11 (accessed 4 Jun 2019).

56 Office for National Statistics. 2011 Census: Key statistics and quick statistics for local authorities in the United Kingdom - part 2. 2014.https://www.ons.gov.uk/employmentandlabourmarket/peopleinwork/employmentandemployeetypes/datasets/2011censuskeystatisticsandquickstatisticsforlocalauthoritiesintheunitedkingdompart2 (accessed 4 Jun 2019).

57 Nomis. Highest level of qualification by sex by age (DC5102EW). 2013.https://www.nomisweb.co.uk/census/2011/dc5102ew (accessed 4 Jun 2019).

58 Nomis. Economic activity by sex by age [DC6107ew]. 2013.https://www.nomisweb.co.uk/census/2011/dc6107ew (accessed 4 Jun 2019).

59 National Institute for Clinical Excellence. Smoking: stopping in pregnancy and after childbirth. 2010.https://www.nice.org.uk/guidance/ph26 (accessed 4 Jun 2019).

For peer review only

For peer review only - http://bmjopen.bmj.com/site/about/guidelines.xhtml

List of Figures:

Figure 1 - Risk of being born Small for Gestational Age (birthweight <10th percentile for gestational age) by parental SES indicators in the University Hospital Southampton (UHS) maternity population-based cohort (singleton live births 2004-2016).

For peer review only

For peer review only - http://bmjopen.bmj.com/site/about/guidelines.xhtml

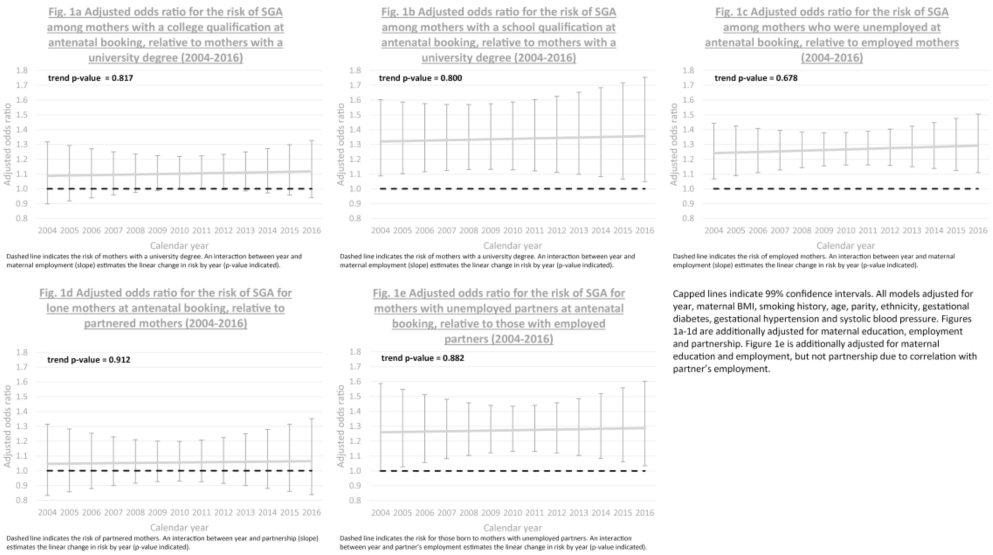

Figure 1 - Risk of being born Small for Gestational Age (birthweight <10th percentile for gestational age) by parental SES indicators in the University Hospital Southampton (UHS) maternity population-based cohort (singleton live births 2004-2016).

338x190mm (300 x 300 DPI)

Supplementary Table 1– Full results of model 4 in Table 2. Risk of being born Small for Gestational Age (birthweight <10th percentile for gestational age) by maternal socioeconomic indicator in the University Hospital Southampton (UHS) maternity population-based cohort (singleton live births 2004-2016).

	OR	99% CI		p
Highest qualification (ref = Degree)				
College qualification	1.104	0.999	1.220	0.011
Secondary school or lower qualification	1.301	1.171	1.445	<0.001
Maternal unemployment	1.267	1.162	1.381	<0.001
Lone mother	1.055	0.930	1.198	0.274
Gestational Diabetes	0.924	0.722	1.182	0.406
Gestational Hypertension	1.928	1.550	2.398	<0.001
Systolic blood pressure >=140 mm Hg	0.999	0.997	1.001	0.447
Multiparous	0.478	0.442	0.517	<0.001
Maternal age at booking	1.013	1.005	1.021	<0.001
Maternal ethnicity (ref = White)				
Mixed	1.300	0.950	1.780	0.031
Asian	2.624	2.291	3.005	<0.001
Black/African/Caribbean	1.788	1.399	2.284	<0.001
Chinese	0.717	0.427	1.204	0.098
Other	1.483	1.123	1.958	<0.001
Not known	1.167	0.998	1.363	0.011
Maternal BMI (ref = Normal weight 18.5-24.9)				
<18.5 (underweight)	1.748	1.479	2.066	<0.001
25-29.9 (overweight)	0.755	0.690	0.826	<0.001
30+ (obese)	0.696	0.622	0.778	<0.001
**Maternal smoking (ref = Never smoked)**				
Ex-smoker	0.948	0.863	1.042	0.145
Up to 10 cigarettes per day	2.248	1.997	2.531	<0.001
10-20 cigarettes per day	2.884	2.504	3.321	<0.001
>20 cigarettes per day	3.780	2.618	5.458	<0.001
Constant	0.133	0.093	0.188	<0.001

n births = 65,412, n mothers = 44,212. OR = odds ratio; CI = confidence interval. In all models the standard errors are adjusted for multiple births per mother.

For peer review only

Supplementary Table 2– Full results of model 4 in Table 3. Risk of being born Small for Gestational Age (birthweight <10th percentile for gestational age) by maternal socioeconomic indicator in the University Hospital Southampton (UHS) maternity population-based cohort (singleton live births 2004-2016).

	OR	99% CI		p
Highest qualification (ref = Degree)				
College qualification	1.094	0.987	1.212	0.024
Secondary school or lower qualification	1.284	1.153	1.431	<0.001
Maternal unemployment	1.234	1.126	1.352	<0.001
Partner unemployment	1.274	1.132	1.434	<0.001
Gestational Diabetes	0.940	0.730	1.210	0.529
Gestational Hypertension	2.005	1.603	2.507	<0.001
Systolic blood pressure >=140 mm Hg	0.999	0.997	1.002	0.497
Multiparous	0.476	0.439	0.516	<0.001
Maternal age at booking	1.014	1.006	1.023	<0.001
Maternal ethnicity (ref = White)				
Mixed	1.270	0.919	1.755	0.057
Asian	2.616	2.278	3.004	<0.001
Black/African/Caribbean	1.716	1.319	2.231	<0.001
Chinese	0.679	0.397	1.162	0.064
Other	1.467	1.100	1.956	0.001
Not known	1.197	1.019	1.406	0.004
Maternal BMI (ref = Normal weight 18.5-24.9)				
<18.5 (underweight)	1.788	1.503	2.127	<0.001
25-29.9 (overweight)	0.755	0.688	0.829	<0.001
30+ (obese)	0.682	0.607	0.767	<0.001
**Maternal smoking (ref = Never smoked)**				
Ex-smoker	0.943	0.856	1.039	0.120
Up to 10 cigarettes per day	2.219	1.959	2.513	<0.001
10-20 cigarettes per day	2.853	2.451	3.322	<0.001
>20 cigarettes per day	3.363	2.225	5.082	<0.001
Constant	0.072	0.051	0.102	<0.001

n births = 61,243, n mothers = 42,265. OR = odds ratio; CI = confidence interval. In all models the standard errors are adjusted for multiple births per mother.

For peer review only

Supplementary Table 3– Risk of being born Small for Gestational Age (birthweight <10th percentile for gestational age) by place of residence and maternal socioeconomic indicator in the University Hospital Southampton (UHS) maternity population-based cohort (singleton live births 2004-2016).

Socioeconomic factor	Sample	OR	99% CI	p
Mothers with a college qualification vs university degree [1]	All	1.10	1.00 - 1.22	0.011
	Southampton	1.10	0.95 - 1.27	0.100
Mothers with a secondary school qualification vs university degree [1]	All	1.30	1.17 - 1.45	<0.001
	Southampton	1.29	1.11 - 1.51	<0.001
Maternal unemployment at the first antenatal appointment vs employed [1]	All	1.27	1.16 - 1.38	<0.001
	Southampton	1.38	1.23 - 1.56	<0.001
Lone motherhood at the first antenatal appointment vs partnered status [1]	All	1.06	0.93 - 1.20	0.274
	Southampton	1.02	0.86 - 1.20	0.805
Mothers with an unemployed partner vs employed partner	All	1.27	1.13 - 1.43	<0.001
	Southampton	1.19	1.03 - 1.39	<0.001

All models adjusted for maternal education, employment, age, ethnicity, parity, gestational diabetes, gestational hypertension and systolic blood pressure. Standard errors are adjusted for multiple births per mother. [1] Also adjusted for maternal partnership. OR = odds ratio; CI = confidence interval.

For peer review only

Supplementary Figure 1 – Sample selection flowchart for the University Hospital Southampton cohort analysis of socioeconomic inequalities in small for gestational age births

Supplementary Figure 1: Sample selection flowchart for the University Hospital Southampton cohort analysis of socioeconomic inequalities in small for gestational age births

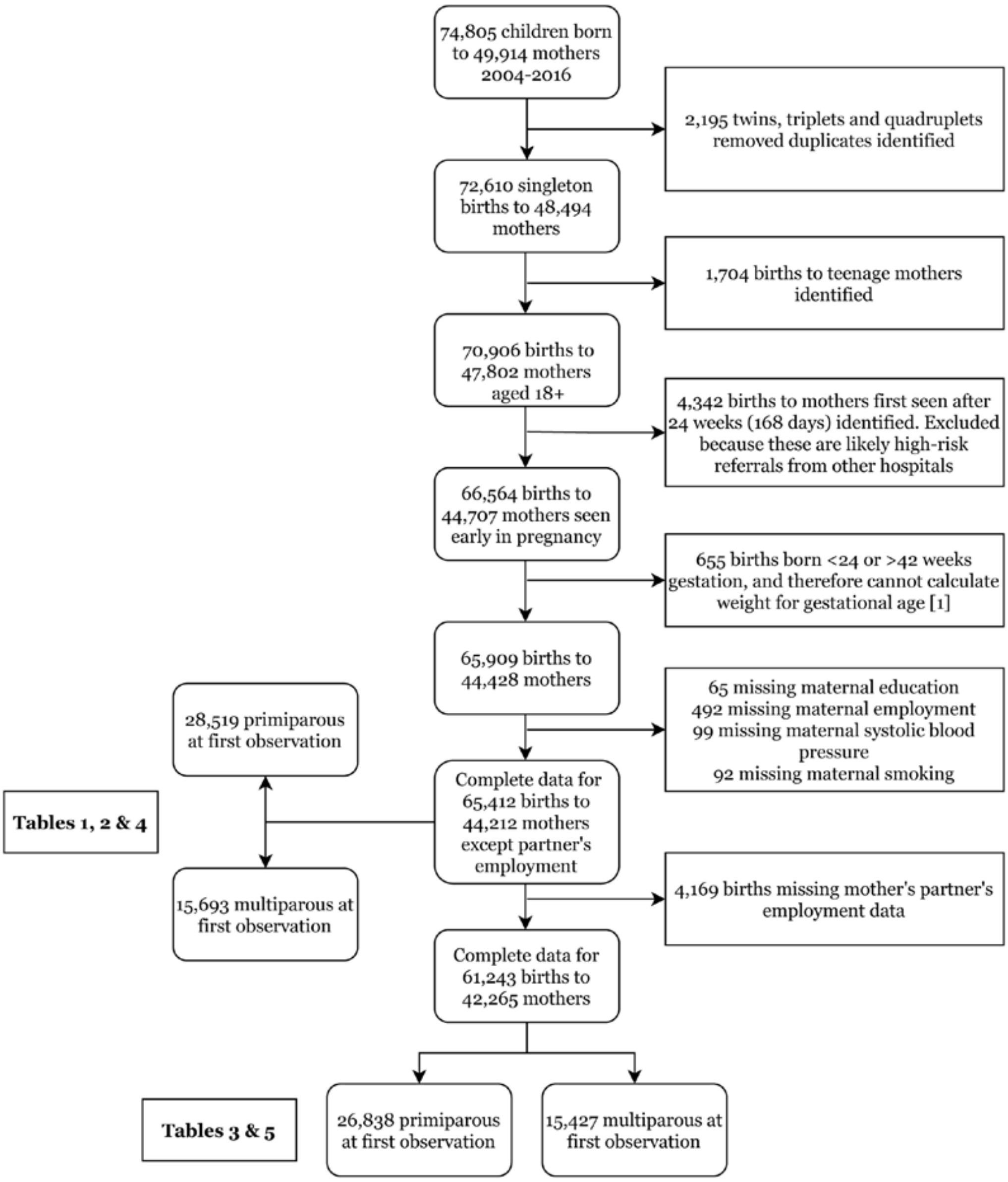

[1] In line with reference data provided in Norris *et al.* Updated birth weight centiles for England and Wales. *Arch Dis Child - Fetal Neonatal Ed.* 2017;103(6):577-582.

Supplementary Figure 2: Trends in socioeconomic factors over time in the University Hospital Southampton cohort analysis of socioeconomic inequalities in small for gestational age births

Supplementary Figure 2a Maternal education by year in cohort

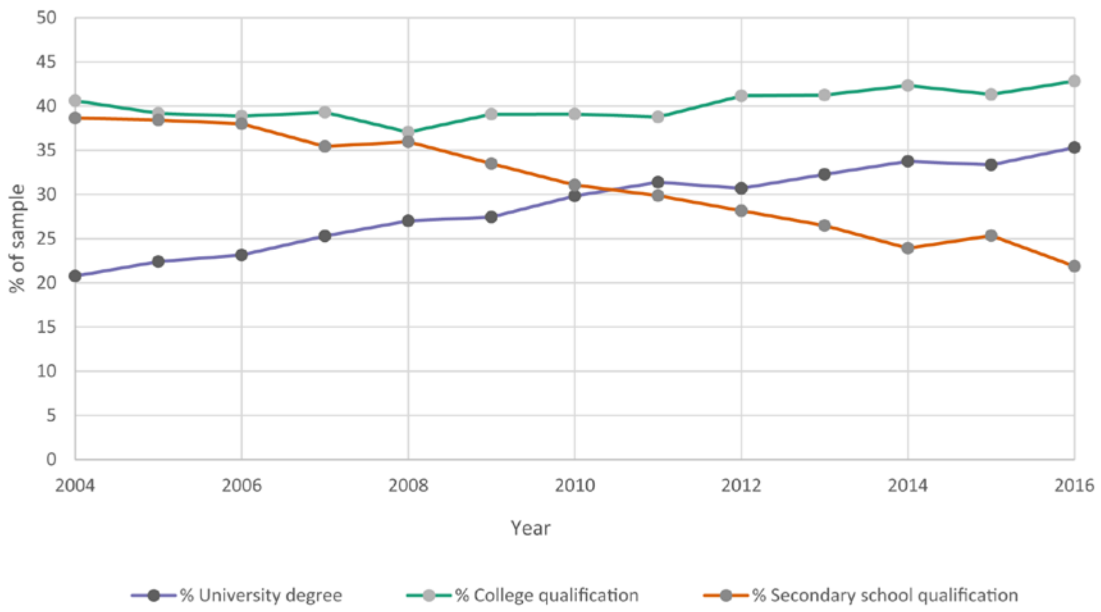

Supplementary Figure 2b Parental employment and partnership by year in cohort

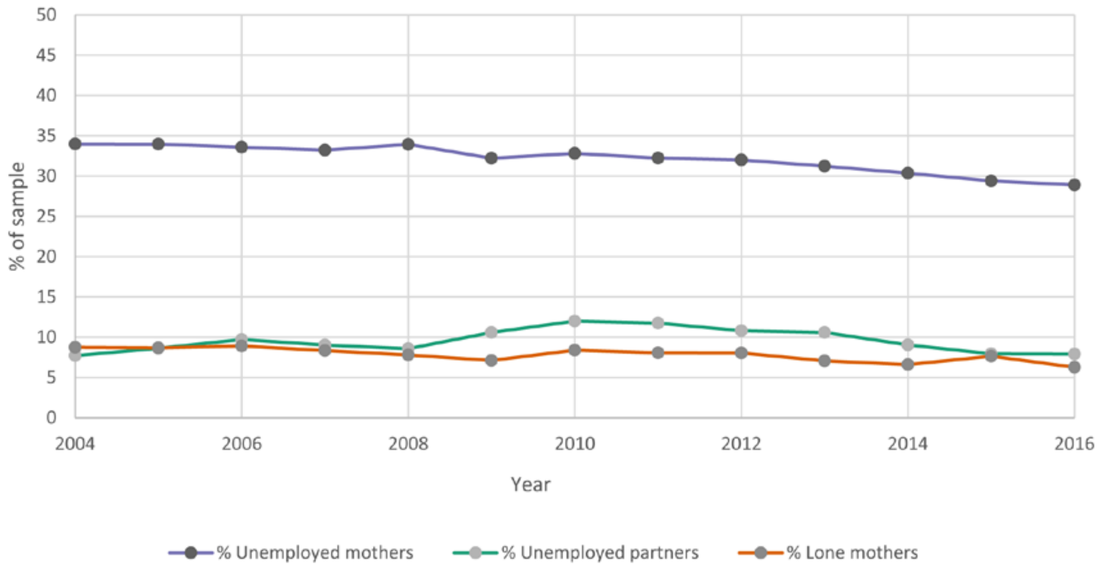

The RECORD statement – checklist of items, extended from the STROBE statement, that should be reported in observational studies using routinely collected health data.

	Item No.	STROBE items	Location in manuscript where items are reported	RECORD items	Location in manuscript where items are reported
**Title and abstract**					
	1	(a) Indicate the study's design with a commonly used term in the title or the abstract<br><br>(b) Provide in the abstract an informative and balanced summary of what was done and what was found	Page 1<br><br>Page 2	RECORD 1.1: The type of data used should be specified in the title or abstract. When possible, the name of the databases used should be included.<br><br>RECORD 1.2: If applicable, the geographic region and timeframe within which the study took place should be reported in the title or abstract.<br><br>RECORD 1.3: If linkage between databases was conducted for the study, this should be clearly stated in the title or abstract.	Page 1<br><br>Page 2<br><br>N/A
**Introduction**					
Background rationale	2	Explain the scientific background and rationale for the investigation being reported	Page 3		
Objectives	3	State specific objectives, including any prespecified hypotheses	Lines 106-110		
**Methods**					
Study Design	4	Present key elements of study design early in the paper	Pages 3-4		
Setting	5	Describe the setting, locations, and relevant dates, including periods of recruitment, exposure, follow-up, and data collection	Pages 4-5		

For peer review only

Participants	6	*(a)* *Cohort study* - Give the eligibility criteria, and the sources and methods of selection of participants. Describe methods of follow-up<br>*Case-control study* - Give the eligibility criteria, and the sources and methods of case ascertainment and control selection. Give the rationale for the choice of cases and controls<br>*Cross-sectional study* - Give the eligibility criteria, and the sources and methods of selection of participants<br><br>*(b)* *Cohort study* - For matched studies, give matching criteria and number of exposed and unexposed<br>*Case-control study* - For matched studies, give matching criteria and the number of controls per case	Page 4; Supplementary Figure 1	RECORD 6.1: The methods of study population selection (such as codes or algorithms used to identify subjects) should be listed in detail. If this is not possible, an explanation should be provided.<br><br>RECORD 6.2: Any validation studies of the codes or algorithms used to select the population should be referenced. If validation was conducted for this study and not published elsewhere, detailed methods and results should be provided.<br><br>RECORD 6.3: If the study involved linkage of databases, consider use of a flow diagram or other graphical display to demonstrate the data linkage process, including the number of individuals with linked data at each stage.	Page 4-6<br><br>N/A<br><br>N/A
Variables	7	Clearly define all outcomes, exposures, predictors, potential confounders, and effect modifiers. Give diagnostic criteria, if applicable.	Page 4-6	RECORD 7.1: A complete list of codes and algorithms used to classify exposures, outcomes, confounders, and effect modifiers should be provided. If these cannot be reported, an explanation should be provided.	Pages 4-6
Data sources/ measurement	8	For each variable of interest, give sources of data and details of methods of assessment (measurement).<br>Describe comparability of assessment methods if there is more than one group	Pages 4-6		

For peer review only - http://bmjopen.bmj.com/site/about/guidelines.xhtml

Bias	9	Describe any efforts to address potential sources of bias	Lines 113-126; 155-169; 181-185; 260-272		
Study size	10	Explain how the study size was arrived at	Page 4; Supplementary Figure 1		
Quantitative variables	11	Explain how quantitative variables were handled in the analyses. If applicable, describe which groupings were chosen, and why	Pages 4-6		
Statistical methods	12	(a) Describe all statistical methods, including those used to control for confounding	Pages 5-6		
		(b) Describe any methods used to examine subgroups and interactions	Pages 5-6		
		(c) Explain how missing data were addressed	Lines 186-188		
		(d) *Cohort study* - If applicable, explain how loss to follow-up was addressed	N/A		
		Case-control study - If applicable, explain how matching of cases and controls was addressed	N/A		
		Cross-sectional study - If applicable, describe analytical methods taking account of sampling strategy	N/A		
		(e) Describe any sensitivity analyses	Lines 260-272		

For peer review only

Data access and cleaning methods		..		RECORD 12.1: Authors should describe the extent to which the investigators had access to the database population used to create the study population.	Page 4
				RECORD 12.2: Authors should provide information on the data cleaning methods used in the study.	N/A
Linkage		..		RECORD 12.3: State whether the study included person-level, institutional-level, or other data linkage across two or more databases. The methods of linkage and methods of linkage quality evaluation should be provided.	N/A
**Results**					
Participants	13	(a) Report the numbers of individuals at each stage of the study (*e.g.*, numbers potentially eligible, examined for eligibility, confirmed eligible, included in the study, completing follow-up, and analysed)<br>(b) Give reasons for nonparticipation at each stage.<br>(c) Consider use of a flow diagram	Page 4; Supplementary Figure 1<br>Page 4; Supplementary Figure 1<br>Supplementary Figure 1	RECORD 13.1: Describe in detail the selection of the persons included in the study (*i.e.*, study population selection) including filtering based on data quality, data availability and linkage. The selection of included persons can be described in the text and/or by means of the study flow diagram.	Pages 4-6; Supplementary Figure 1

For peer review only

Descriptive data	14	(a) Give characteristics of study participants (*e.g.*, demographic, clinical, social) and information on exposures and potential confounders<br>(b) Indicate the number of participants with missing data for each variable of interest<br>(c) *Cohort study* - summarise follow-up time (*e.g.*, average and total amount)	Table 1<br><br>Table 1<br><br>N/A		
Outcome data	15	*Cohort study* - Report numbers of outcome events or summary measures over time<br>*Case-control study* - Report numbers in each exposure category, or summary measures of exposure<br>*Cross-sectional study* - Report numbers of outcome events or summary measures	Table 1, Supplementary Figure 2<br><br>N/A<br><br>N/A		
Main results	16	(a) Give unadjusted estimates and, if applicable, confounderadjusted estimates and their precision (e.g., 95% confidence interval). Make clear which confounders were adjusted for and why they were included<br>(b) Report category boundaries when continuous variables were categorized<br>(c) If relevant, consider translating estimates of relative	Tables 2-5; Page 5<br><br>Table 1<br><br>N/A		

For peer review only

		risk into absolute risk for a meaningful time period			
Other analyses	17	Report other analyses done—e.g., analyses of subgroups and interactions, and sensitivity analyses	Tables 4-5; lines 260-272		
**Discussion**					
Key results	18	Summarise key results with reference to study objectives	Page 18		
Limitations	19	Discuss limitations of the study, taking into account sources of potential bias or imprecision. Discuss both direction and magnitude of any potential bias	Pages 19-20	RECORD 19.1: Discuss the implications of using data that were not created or collected to answer the specific research question(s). Include discussion of misclassification bias, unmeasured confounding, missing data, and changing eligibility over time, as they pertain to the study being reported.	Pages 19-20
Interpretation	20	Give a cautious overall interpretation of results considering objectives, limitations, multiplicity of analyses, results from similar studies, and other relevant evidence	Pages 19-20		

For peer review only

Generalisability	21	Discuss the generalisability (external validity) of the study results	Lines 418-435		
**Other Information**					
Funding	22	Give the source of funding and the role of the funders for the present study and, if applicable, for the original study on which the present article is based	Lines 488-493		
Accessibility of protocol, raw data, and programming code		..		RECORD 22.1: Authors should provide information on how to access any supplemental information such as the study protocol, raw data, or programming code.	Lines 514-519

*Reference: Benchimol EI, Smeeth L, Guttmann A, Harron K, Moher D, Petersen I, Sørensen HT, von Elm E, Langan SM, the RECORD Working Committee. The REporting of studies Conducted using Observational Routinely-collected health Data (RECORD) Statement. *PLoS Medicine* 2015; in press.

*Checklist is protected under Creative Commons Attribution (CC BY) license.

For peer review only
